# Applying survey weights to ordinal regression models for improved inference in outcome-dependent samples with ordinal outcomes

**DOI:** 10.1177/09622802241282091

**Published:** 2024-10-23

**Authors:** Aya A Mitani, Osvaldo Espin-Garcia, Daniel Fernández, Victoria Landsman

**Affiliations:** 1 Dalla Lana School of Public Health, University of Toronto, ON, Canada; 2 Department of Epidemiology and Biostatistics, Western University, ON, Canada; 3 Department of Statistical Sciences, University of Toronto, ON, Canada; 4 Department of Biostatistics & Schroeder Arthritis Institute, University Health Network, ON, Canada; 5 Serra Húnter fellow. Department of Statistics and Operations Research, Universitat Politècnica de Catalunya - BarcelonaTech, Barcelona, Spain; 6 Institute for Work and Health, ON, Canada

**Keywords:** biased sampling, inverse sampling weights, proportional odds model, stereotype model, cumulative logit, adjacent-category logit, continuation-ratio logit, knee osteoarthritis

## Abstract

Researchers often use outcome-dependent sampling to study the exposure-outcome association. The case-control study is a widely used example of outcome-dependent sampling when the outcome is binary. When the outcome is ordinal, standard ordinal regression models generally produce biased coefficients when the sampling fractions depend on the values of the outcome variable. To address this problem, we studied the performance of survey-weighted ordinal regression models with weights inversely proportional to the sampling fractions. Through an extensive simulation study, we compared the performance of four ordinal regression models (SM: stereotype model; AC: adjacent-category logit model; CR: continuation-ratio logit model; and CM: cumulative logit model), with and without sampling weights under outcome-dependent sampling. We observed that when using weights, all four models produced estimates with negligible bias of all regression coefficients. Without weights, only stereotype model and adjacent-category logit model produced estimates with negligible to low bias for all coefficients except for the intercepts in all scenarios. In one scenario, the unweighted continuation-ratio logit model also produced estimates with low bias. The weighted stereotype model and adjacent-category logit model also produced estimates with lower relative root mean square errors compared to the unweighted models in most scenarios. In some of the scenarios with unevenly distributed categories, the weighted continuation-ratio logit model and cumulative logit model produced estimates with lower relative root mean square errors compared to the respective unweighted models. We used a study of knee osteoarthritis as an example.

## 1. Introduction

Outcome-dependent sampling (ODS) or disease-based sampling is commonly implemented in observational studies when the outcome prevalence is low or when study resources are limited.^1–3^ Under ODS, a subset of participants from an existing cohort is retrospectively sampled with a sampling probability that depends on their observed value of the outcome variable. Then, the expensive exposure variable is ascertained only for the new subsample of participants. It is well established that ODS can be more efficient than simple random sampling (SRS).^4^ When the outcome of interest is a binary variable, then the ODS design is equivalent to a case-control study, and logistic regression is the standard form of analysis. Despite the oversampling of cases that occurs in case-control studies, the logistic regression coefficient estimates obtained from the sampled data are consistent for the population, except for the intercept.^5,6^

Ordinal data are categorical data where the categories possess a meaningful order, distinguishing them from nominal data with no inherent ordering. These variables commonly arise in pain or disease severity scales in health assessments. Despite their prevalence, most existing methods for analyzing ordinal data treat them as nominal or continuous, neglecting the unique characteristics of ordinal outcomes.^7^ Nevertheless, adopting ordinal-specific statistical models can be more advantageous.^8^ In this article, we are interested in exploring statistical methods to model ordinal categorical outcomes under ODS. To the best of our knowledge, our work represents the first comprehensive exploration of regression models specifically tailored to model ordinal categorical outcomes under ODS. Examples of ordinal outcomes in this framework are the Global Clinical Impression Scale to assess the symptoms and functional ability of participants with mental disorders, the Glasgow outcome scale to assess the functional outcomes of participants with brain injury, and the BI-RADS density scale to classify breast density.^9,10^ If the exposure of interest, such as a new genetic marker from stored sample specimens or new readings from stored computed tomography (CT) scan images, needs to be ascertained retrospectively, and if the ascertainment is costly, then ODS will generally result in more statistically efficient estimates with smaller standard errors than SRS.^11,12^ Our illustrative example comes from a knee osteoarthritis (OA) study, where the severity of the disease is expressed using the Kellgren-Lawrence (KL) radiographic grade that ranges from 0 (absence of OA) to 4 (most severe form of OA). To study the association between KL grade and a genetic marker among knee OA patients in a resource-limited setting, an ODS framework may be advantageous for researchers.

The ordinal regression model with various logits (AC: adjacent-category logit; CR: continuation-ratio logit; CM: cumulative logit) assuming proportional odds is a well-known and commonly used extension of logistic regression to ordinal outcomes. However, the regression coefficient estimates obtained from CM and CR under ODS are biased.^13,14^ Greenland^13^ further showed that the regression coefficients (except for the intercept) obtained from the stereotype model (SM) under ODS are consistent estimators of the original cohort. However, if a pre-specified number of participants are randomly sampled from each outcome category, then an ordinal ODS design is equivalent to stratified random sampling where the strata are defined by the categories of the outcome of interest. Design-consistent inference about the population from a stratified sample can be obtained by weighing each observation with the inverse of its inclusion (or sampling) probability. Therefore, if the sampling probability is known by design, then any ordinal regression model weighted by the inverse of the sampling probability should produce consistent coefficient estimates including the intercepts. Although the accuracy of the intercept is often overlooked under ODS, it might be important if the interest lies in estimating the absolute risks or risk differences.

The objective of this study is to compare the bias and efficiency of the estimated regression coefficients from weighted and unweighted ordinal regression models (SM, AC, CR, and CM) in ordinal ODS under various distributions of outcome categories. For weighted models, we obtain design-based standard error estimates using linearization^15^ and provide code to apply linearization to SM estimated from the VGAM package^16^ in the statistical software R.^17^ The plan of the article is as follows. In the following section, we review the four ordinal regression models and describe the implications of using each of them under ODS. In Section 4, we describe our simulation study and present the results. In Section 5, we compare the results between the four models fit to ODS of a real cohort of participants with knee OA. Finally, in Section 6, we give some concluding remarks and directions for future research.

## 2. Review of ordinal regression models

Let $Y_{i}$ be the ordinal outcome variable for the i th subject that takes values k=1,…,K , with $\mathrm{Pr}(Y_{i}=k)=\pi_{ik}$ assumed to follow a multinomial distribution. Associated with each $Y_{i}$ is a set of p covariates $X_{i}=(X_{i1},\ldots ,X_{ip}{)}^{\prime }$ . In general, each ordinal regression model relates the probabilities $\pi_{ik}$ to $\alpha_{k}+\beta^{\prime }X_{i}$ through a logit link function as described below. Here, $\alpha_{k}$ is the intercept term for the k th category and β is the vector of regression coefficients for $X_{i}$ that has a unique interpretation for each type of logit link function. The proportional odds assumption is often used when applying ordinal regression models in which the effects of each $\beta_{j}$ in $\beta =(\beta_{1},\ldots ,\beta_{p}{)}^{\prime }$ are the same for each logit, which makes the model more parsimonious.^7^

### 2.1. Stereotype model

The stereotype model (SM) for ordinal outcomes was proposed by Anderson^18^ and is considered a more general form of the proportional odds model.^7^ SM uses a baseline-category logit and additional score parameters, $\phi_{1},\phi_{2},\ldots ,\phi_{K}$ . Incorporation of score parameters enhances the flexibility of SM compared to the CM proportional odds model (Agresti,^7^ Section 4.3.4).

The model is described as follows:

(1) $$\ln\;\frac{\pi_{ik}}{\pi_{iK}}=\alpha_{k}+\phi_{k}\beta^{\prime }X_{i},\;k=1,\ldots ,K,$$

with $\alpha_{K}=\phi_{K}=0$ . To uniquely identify the parameters, we also impose the restriction $\phi_{1}=1$ . Therefore, the number of parameters to be estimated is (K−1)+(K−2)+p=2K−3+p . With these identifiability constraints, the category-specific probabilities are

(2) $$\pi_{ik}=\frac{\text{exp}(\alpha_{k}+\phi_{k}\beta^{\prime }X_{i})}{1+\sum_{h=1}^{K-1}\text{exp}(\alpha_{h}+\phi_{h}\beta^{\prime }X_{i})},\;k=1,\ldots ,K-1$$

and $\pi_{iK}=1-\sum_{h=1}^{K-1}\pi_{h}$ . In SM, $\phi_{k}\beta_{j}$ for the i th subject represents the change in the log odds ratio of observing $Y_{i}=k$ versus $Y_{i}=K$ per unit increase in $X_{ij}$ , assuming $X_{ij}$ to be numerical, for j=1,…,p . Score parameters can be fixed or estimated from the data.^13^ The distance between two adjacent score parameters, defined as $\phi_{k+1}-\phi_{k}$ , is related to the similarity between $Y_{k+1}$ and $Y_{k}$ . If $\phi_{k+1}-\phi_{k}=0$ , then the covariates $X_{i}$ provide no evidence to distinguish between these two categories. Therefore, we can conduct a hypothesis test with the null hypothesis: $\phi_{k}=\phi_{k+1}$ , to determine whether adjacent categories k and k+1 are indistinguishable, according to the data. If the null hypothesis is not rejected, then categories k and k+1 can be collapsed into a single outcome category^7,19^ to render a simpler model.

### 2.2. Adjacent-category model

The adjacent-category (AC) models the log odds of two consecutive categories and has the form

(3) $$\ln\;\frac{\pi_{ik}}{\pi_{ik+1}}=\alpha_{k}+\beta^{\prime }X_{i},\;k=1,\ldots ,K-1$$

In AC, $\beta_{j}$ represents the change in the log odds of two adjacent categories, $\pi_{ik}$ to $\pi_{ik+1}$ per unit increase in $X_{ij}$ , assuming $X_{ij}$ to be numerical, for each j=1,…,p . The number of parameters to be estimated is K+p−1 . AC is useful when there is a meaningful relationship or progression between the adjacent categories. The category-specific probabilities are

$$\pi_{ik}=\frac{\exp\;(\sum_{h=k}^{K-1}\alpha_{h}+\beta^{\prime }X_{i})}{1+\sum_{h=1}^{K-1}\exp\;(\sum_{l=h}^{K-1}\alpha_{l}+\beta^{\prime }X_{i})},\;k=1,\ldots ,K-1$$

and $\pi_{iK}=1-\sum_{h=1}^{K-1}\pi_{h}$ .

AC can be re-expressed as SM with fixed and equally spaced score parameters:^7^

(4) $$\begin{array}{rl} \ln\;\frac{\pi_{ik}}{\pi_{iK}} & =\ln\;\left(\frac{\pi_{ik}}{\pi_{ik+1}}\times \frac{\pi_{ik+1}}{\pi_{ik+2}}\times \cdots \times \frac{\pi_{iK-1}}{\pi_{iK}}\right)\\ & =\ln\;\frac{\pi_{ik}}{\pi_{ik+1}}+\ln\;\frac{\pi_{ik+1}}{\pi_{ik+2}}+\cdots \ln\;\frac{\pi_{iK-1}}{\pi_{iK}}\\ & =\sum_{h=k}^{K-1}\alpha_{h}+(K-k)\beta^{\prime }X_{i},\;k=1,\ldots ,K-1 \end{array}$$

which is equivalent to SM with intercept equal to $\sum_{h=k}^{K-1}\alpha_{h}$ and score parameters equal to K−k for each k=1,…K−1 . SM and AC are paired-category logit models where the logit function involves a pair of probabilities as opposed to the continuation-ratio and CM models described below.

### 2.3. CR Continuation-ratio modelmodel

The continuation-ratio (CR) model is

(5) $$\ln\;\frac{\pi_{ik}}{\pi_{ik+1}+\cdots +\pi_{iK}}=\alpha_{k}+\beta^{\prime }X_{i},\;k=1,\ldots ,K-1$$

In CR, $\beta_{j}$ represents the change in the log odds of being in category k to being in all the categories above k per unit increase in $X_{ij}$ , assuming $X_{ij}$ to be numerical, for each j=1,…,p . The number of parameters to be estimated is K+p−1 . CR is useful when the categories represent a sequence of levels in which an individual progresses from the lowest to the highest level. Examples are individual’s survival through various age groups and individual’s length of hospital stay from short to long.^20^ The category-specific probabilities are

(6) $$\pi_{ik}=\frac{\exp\;(\alpha_{k}+\beta^{\prime }X_{i})}{\prod_{h=1}^{k}\left\{1+\exp\;(\alpha_{h}+\beta^{\prime }X_{i})\right\}},\;k=1,\ldots ,K-1$$

and $\pi_{iK}=1-\sum_{h=1}^{K-1}\pi_{ih}$ .

### 2.4. Cumulative logit model

The cumulative logit (CM) model, often referred to as “the proportional odds logistic regression,” is the most widely used model when the outcome variable has more than two categories, and is useful when the interest is in modelling whether patients improved in disease category based on a treatment. CM is given by the following:

(7) $$\ln\;\frac{\pi_{i1}+\cdots +\pi_{ik}}{\pi_{ik+1}+\cdots +\pi_{iK}}=\alpha_{k}+\beta^{\prime }X_{i},\;k=1,\ldots ,K-1$$

In CM, $\beta_{j}$ represents the change in the log odds ratio of observing $Y_{i}\leq k$ to $Y_{i}>k$ per unit increase in $X_{ij}$ , assuming $X_{ij}$ to be numerical, for each category k and for each j=1,…,p . The number of parameters to be estimated is K+p−1 , same as for AC and CR models. The category-specific probabilities are

(8) $$\begin{array}{rl} \pi_{i1} & =\frac{\exp\;(\alpha_{1}+\beta^{\prime }X_{i})}{1+\exp\;(\alpha_{1}+\beta^{\prime }X_{i})}\\ \pi_{ik} & =\frac{\exp\;(\alpha_{k}+\beta^{\prime }X_{i})}{1+\exp\;(\alpha_{k}+\beta^{\prime }X_{i})}-\frac{\exp\;(\alpha_{k-1}+\beta^{\prime }X_{i})}{1+\exp\;(\alpha_{k-1}+\beta^{\prime }X_{i})},\;k=2,\ldots ,K-1 \end{array}$$

and $\pi_{iK}=1-\sum_{h=1}^{K-1}\pi_{ih}$ .

## 3. Ordinal regression models under ODS

Suppose now that the original cohort consists of N individuals that can be divided into K ordered outcome categories. Each outcome category consists of $N_{k}$ individuals for k=1,2,…,K , and $N=\sum_{k=1}^{K}N_{k}$ . We want to study the association between the increasing odds of the outcome and an unmeasured exposure. Due to limited resources, the unmeasured exposure cannot be ascertained from all N individuals in the cohort. Therefore, we sample $n_{k}$ individuals from each outcome category, where $n_{k}<N_{k}$ . Let $S_{i}$ be the sampling indicator equal to 1 if subject i is included in the sample S and 0 otherwise. By design, the sampling probability of i∈S is conditionally independent of the covariates given the observed outcome value for i , that is, $\mathrm{Pr}(S_{i}=1|Y_{i}=k,X_{i})=\mathrm{Pr}(S_{i}=1|Y_{i}=k)$ . Therefore, the sampling probability of i can be expressed as $f_{ik}=\mathrm{Pr}(S_{i}=1|Y_{i}=k)=(n_{k}/N_{k}):=f_{k}$ for each k=1,…,K . We then ascertain the unmeasured exposure for all i∈S and estimate the association between the newly ascertained exposure and the increasing odds of the outcome.

When K=2 and $N_{1}$ and $N_{2}$ are diseased and disease-free individuals, respectively, this design is a (nested) case-control study.^21^ We typically use logistic regression to estimate the association between the binary outcome and exposure of a case-control sample. A well-known property of the logistic regression model is that the coefficient for the exposure is a consistent estimator of the original cohort, but the intercept term is biased by $\ln\;(f_{2}/f_{1})$ .^6^ When K>2 and there is an intrinsic order in the outcome, ordinal regression models are the typical choice of analysis. In the rest of this section, we describe the implications of using each of the four ordinal regression models (SM, AC, CR, and CM) reviewed in the previous sections.

Let $\pi_{ik}^{s}=\mathrm{Pr}(Y_{i}=k|X_{i},S_{i}=1)$ be the category-specific probability of i∈S for i=1,…,n where $n=\sum_{k=1}^{K}n_{k}$ . Under the assumption that the sampling probability is conditionally independent of the covariates given the observed outcome value for an individual i , $\pi_{ik}^{s}$ can be expressed as follows for any ordinal regression model by applying the Bayes theorem:

(9) $$\begin{array}{rl} \pi_{ik}^{s} & =\mathrm{Pr}(Y_{i}=k|X_{i},S_{i}=1)\\ & =\frac{\mathrm{Pr}(S_{i}=1|Y_{i}=k,X_{i})\mathrm{Pr}(Y_{i}=k|X_{i})}{\sum_{h=1}^{K}\left\{\mathrm{Pr}(S_{i}=1|Y_{i}=h,X_{i})\mathrm{Pr}(Y_{i}=h|X_{i})\right\}}\\ & =\frac{\mathrm{Pr}(S_{i}=1|Y_{i}=k)\mathrm{Pr}(Y_{i}=k|X_{i})}{\sum_{h=1}^{K}\left\{\mathrm{Pr}(S_{i}=1|Y_{i}=h)\mathrm{Pr}(Y_{i}=h|X_{i})\right\}}\\ & =\frac{f_{k}\mathrm{Pr}(Y_{i}=k|X_{i})}{\sum_{h=1}^{K}\left\{f_{h}\mathrm{Pr}(Y_{i}=h|X_{i})\right\}}\\ & =\frac{f_{k}\pi_{ik}}{\sum_{h=1}^{K}\left\{f_{h}\pi_{ih}\right\}} \end{array}$$

If we sample the same proportion of individuals from each outcome category ( $f_{k}=n_{k}/N_{k}=n/N$ for all k=1,…,K ), which corresponds to proportional allocation in stratified random sampling,^22^ then $\pi_{ik}^{s}=\pi_{ik}$ for all four models. However, under ODS with unequal $f_{k}$ ’s , the relationship between $\pi_{ik}^{s}$ and $\pi_{ik}$ depends on the specific version of the ordinal regression model. For SM, by plugging in equation (2) into equation (9), we obtain

(10) $$\pi_{ik}^{s}=\frac{\exp\;(\alpha_{k}^{s}+\phi_{k}\beta^{\prime }X_{i})}{1+\sum_{h=1}^{K-1}\exp\;(\alpha_{h}^{s}+\phi_{h}\beta^{\prime }X_{i})},\;k=1,\ldots ,K-1$$

where $\alpha_{k}^{s}=\alpha_{k}+\ln\;(f_{k}/f_{K})$ and $\alpha_{h}^{s}=\alpha_{h}+\ln\;(f_{h}/f_{K})$ and $\pi_{iK}^{s}=1-\sum_{h=1}^{K-1}\pi_{ih}^{s}$ .

For AC, we obtain

(11) $$\pi_{ik}^{s}=\frac{\exp\;(\alpha_{h}^{s}+\beta^{\prime }X_{i})}{1+\sum_{l=1}^{K-1}\exp\;(\alpha_{l}^{s}+\beta^{\prime }X_{i})}$$

where $\alpha_{h}^{s}=\sum_{h=k}^{K-1}\alpha_{k}+\ln\;(f_{k}/f_{K})$ and $\alpha_{l}^{s}=\sum_{l=h}^{K-1}\alpha_{h}+\ln\;(f_{h}/f_{K})$ for k=1,…,K−1 , and $\pi_{iK}^{s}=1-\sum_{h=1}^{K-1}\pi_{ih}^{s}$ . The full derivations of equations (10) and (11) are given in Appendix I.

Equation (10) implies that, in SM, the β and $\phi_{k}$ coefficients in $\pi_{ik}^{s}$ are not dependent on S , but $\alpha_{k}^{s}$ is offset by $\ln\;\left(f_{k}/f_{K}\right)$ for each k=1,…,K ; and similarly for AC as implied by equation (11). On the other hand, for CR and CM, equation (9) cannot be expressed in the same form as equations (6) and (8), respectively,^13^ except in the special case when $f_{2}=f_{3}=\cdots =f_{K}$ for CR (Appendix II).

### 3.1. Estimation

The log-likelihood contribution from the i th individual is given by $\ell_{i}(\theta )=\sum_{k=1}^{K}I(Y_{i}=k)\ln\;\mathrm{Pr}(Y_{i}=k|X_{i};\theta )$ , where I(⋅) is the indicator function and θ is the vector of parameters. For SM, $\theta =(\alpha^{\prime },\phi^{\prime },\beta^{\prime }{)}^{\prime }$ , $\alpha =(\alpha_{1},\ldots ,\alpha_{K-1}{)}^{\prime }$ , $\phi =(\phi_{2},\ldots ,\phi_{K-1}{)}^{\prime }$ and for CM, CR, and AC, $\theta =(\alpha^{\prime },\beta^{\prime }{)}^{\prime }$ .

Under ODS, we obtain design-consistent estimates of the parameters in the ordinal regression models by maximizing the log of the pseudo-likelihood

(12) $$\ell_{w}(\theta )=\sum_{i=1}^{n}w_{i}\ell_{i}(\theta ),$$

where the weights $w_{i}=w_{i}(k)={f_{k}}^{-1}$ are defined as the inverse of the sample inclusion probability for the i th observation given that their outcome value is k , for i∈S and k=1,…,K . Throughout the rest of the article, we refer to the value that maximizes $\ell_{w}(\theta )$ as the weighted estimator, ${\hat{\theta }}_{w}$ . If $w_{i}(k)=w$ for each k=1,…,K , which is the case of SRS, then the value that maximizes equation (12) is the usual maximum likelihood estimator, ${\hat{\theta }}_{u}$ .

To construct design-based standard error estimates of ${\hat{\theta }}_{w}$ , we relied on the asymptotic linearity of a weighted estimator^23^

$$\sqrt{n}({\hat{\theta }}_{w}-\theta )=\sum_{i=1}^{n}w_{i}z_{i}(\theta )+o_{p}(n^{-1/2}),$$

where $z_{i}(\theta )={\hat{I}}_{iw}^{-1}U_{i}$ is the influence function of the i th individual with $U_{i}=\frac{\partial }{\partial \theta }\ell_{i}(\theta )$ , the score component of the i th individual and ${\hat{I}}_{iw}=-\sum_{i}\frac{\partial^{2}}{\partial \theta^{2}}w_{i}\ell_{i}(\theta )$ , the observed Fisher information of the pseudo-likelihood.^24^ Then, the sampling variance of ${\hat{\theta }}_{w}$ can be approximated by the sampling variance of the weighted sum of the influence functions.^15^

We used the VGAM package in R^16^ to fit all ordinal regression models. Specifically, we used the rrvglm() function to fit SM and the rvglm() function to fit AC, CR, and CM. For the weighted AC, CR, and CM models that were fit using the rvglm() function with the weights option, we used the svyVGAM package^25^ to obtain design-based standard error estimates, which uses the influence function approach described above. However, a corresponding wrapper function for rrvglm() is not available in the svyVGAM package. Therefore, we developed our own R function to obtain design-based estimates from rrvglm() models by incorporating the svytotal() function in the survey package.^26^ The function is available from https://github.com/ayamitani/svy\_sm. For SM, in addition to the individual parameter estimates in θ , the log odds estimates ${\hat{\phi }}_{k}\hat{\beta }$ , for k=1,…,K−1 are also of interest. To obtain design-based standard error estimates of ${\hat{\phi }}_{k}\hat{\beta }$ , we applied the delta method.

## 4. Simulation study

### 4.1. Design

We conducted an extensive simulation study to compare the bias and efficiency of unweighted ( ${\hat{\theta }}_{u}$ ) and weighted ( ${\hat{\theta }}_{w}$ ) estimators from the four ordinal regression models (SM, AC, CR, and CM) under ODS. For each of the 1000 simulations, we generated a cohort of N=10,000 individuals that can be divided into three ( K=3 ) or five ( K=5 ) disease categories from various distributions. Within each value of K , we varied the proportions of the lowest (Scenario L), middle (Scenario M), and highest (Scenario H) categories while distributing the proportions of the remaining categories evenly (Table 1). We considered two independent predictors: a continuous predictor $X_{1i}$ was generated from a continuous uniform distribution with support (0,1) and a binary predictor $X_{2i}$ was generated from a binomial distribution with probability 0.5.

**Table 1.** Proportions in each of the ordinal outcome categories to generate the full cohort in each simulation scenario (same for A1 and A2) and mean CV of the inverse probability weights from ODS (A1).

	K=3		K=5	
Scenario	Pr(Y=k),k=1,…,K	CV	Pr(Y=k),k=1,…,K	CV
L(i)	0.1 0.45 0.45	0.46	0.04 0.24 0.24 0.24 0.24	0.38
L(ii)	0.3 0.35 0.35	0.06	0.2 0.2 0.2 0.2 0.2	0.07
L(iii)	0.5 0.25 0.25	0.36	0.4 0.15 0.15 0.15 0.15	0.52
L(iv)	0.7 0.15 0.15	0.76	0.6 0.1 0.1 0.1 0.1	0.98
L(v)	0.9 0.05 0.05	1.17	0.8 0.05 0.05 0.05 0.05	1.46
M(i)	0.45 0.1 0.45	0.51	0.24 0.24 0.04 0.24 0.24	0.41
M(ii)	0.35 0.3 0.35	0.11	0.2 0.2 0.2 0.2 0.2	0.07
M(iii)	0.25 0.5 0.25	0.29	0.15 0.15 0.4 0.15 0.15	0.44
M(iv)	0.15 0.7 0.15	0.71	0.1 0.1 0.6 0.1 0.1	0.92
M(v)	0.05 0.9 0.05	1.15	0.05 0.05 0.8 0.05 0.05	1.42
H(i)	0.45 0.45 0.1	0.46	0.24 0.24 0.24 0.24 0.04	0.38
H(ii)	0.35 0.35 0.3	0.06	0.2 0.2 0.2 0.2 0.2	0.07
H(iii)	0.25 0.25 0.5	0.36	0.15 0.15 0.15 0.15 0.4	0.52
H(iv)	0.15 0.15 0.7	0.75	0.1 0.1 0.1 0.1 0.6	0.99
H(v)	0.05 0.05 0.9	1.17	0.05 0.05 0.05 0.05 0.8	1.46

CV: coefficient of variation; ODS: outcome-dependent sampling.

The coefficients in each SM, AC, CR, and CM have different interpretations and cannot be directly compared to each other. Therefore, we generated ordinal outcomes from four separate models: SM, AC, CM, and CR. However, we wanted the data generated from each model to be comparable in outcome and covariate distributions as well as the outcome-covariate relationship. Therefore, the values of the true vector θ for each model were determined by fitting each of the four models to data generated from the following ordinal probit model with 1 million observations.

(13) $$\mathrm{Pr}(Y_{i}\leq k|X_{i})=\Phi (\alpha_{k}^{*}-1X_{1i}+0.5X_{2i}),\;k=1,\ldots ,K-1$$

The values of $\alpha_{k}^{*}$ varied according to K and the proportion of individuals in each outcome category presented in Table 1. The values of the true vector θ for each of the model used to simulate the cohort are shown in Tables S1 to S6 in the Supplemental Material. The true values for ϕ in SM were (1, 0.5, 0) for K=3 and (1, 0.75, 0.5, 0.25, 0) for K=5 regardless of the scenario.

For each of the four models, we simulated a cohort of N=10,000 individuals and used ODS to sample $n_{k}=100$ (for K=3 ) and $n_{k}=80$ (for K=5 ) individuals from each outcome category, k=1,…,K . We then obtained ${\hat{\theta }}_{u}$ and ${\hat{\theta }}_{w}$ along with their standard error estimates by fitting each corresponding model to the ODS sample. We repeated the simulation 1000 times. To assess the biases of ${\hat{\theta }}_{u}$ and ${\hat{\theta }}_{w}$ , we computed the percent mean relative biases within each model from 1000 simulations. The percent mean relative biases were defined as RB( ${\hat{\theta }}_{u}$ ) = $\frac{1}{1000}\sum_{r=1}^{1000}\left({\hat{\theta }}_{u}^{\left(r\right)}/\theta -1\right)\times 100\%$ and RB( ${\hat{\theta }}_{w}$ ) = $\frac{1}{1000}\sum_{r=1}^{1000}\left({\hat{\theta }}_{w}^{\left(r\right)}/\theta -1\right)\times 100\%$ . To assess the standard error estimates, we computed the percent relative error in model-based standard error for the unweighted model, $\text{RE}({\hat{\theta }}_{u})=\left(\frac{\hat{\text{SE}}({\hat{\theta }}_{u})}{\text{ESE}({\hat{\theta }}_{u})}-1\right)\times 100\%$ and the percent relative error in design-based standard error for the weighted model, $\text{RE}({\hat{\theta }}_{w})=\left(\frac{\hat{\text{SE}}({\hat{\theta }}_{w})}{\text{ESE}({\hat{\theta }}_{w})}-1\right)\times 100\%$ . $\hat{\text{SE}}({\hat{\theta }}_{u})$ was defined as the mean of the model-based standard error estimates from the unweighted model, $\hat{\text{SE}}({\hat{\theta }}_{w})$ was defined as the mean of the design-based standard error estimates from the weighted model, and ESE() was defined as the standard deviation of the ${\hat{\theta }}_{u/w}^{\left(r\right)}$ across r=1,…,1000 simulations. For SM, we additionally estimated the log odds and their standard error estimates from the unweighted $({\hat{\phi }}_{k}\hat{\beta }{)}_{u}$ and weighted $({\hat{\phi }}_{k}\hat{\beta }{)}_{w}$ models for k=2,…,K−1 . To assess efficiency, we computed the relative root mean squared error (RRMSE) between ${\hat{\beta }}_{u}$ and ${\hat{\beta }}_{w}$ from 1000 simulations within each of the four models. RRMSE was defined as $\frac{{\text{RMSE}}_{w}}{{\text{RMSE}}_{u}}=\sqrt{\frac{1}{1000}\sum_{r=1}^{1000}{\left({\hat{\beta }}_{w}^{\left(r\right)}-\beta \right)}^{2}}/\sqrt{\frac{1}{1000}\sum_{r=1}^{1000}{\left({\hat{\beta }}_{u}^{\left(r\right)}-\beta \right)}^{2}}$ .

The R statistical environment was used to conduct the simulation study. Packages and functions used to fit each model are described in Section 3.1.

### 4.2. Results

**Figure 1.**
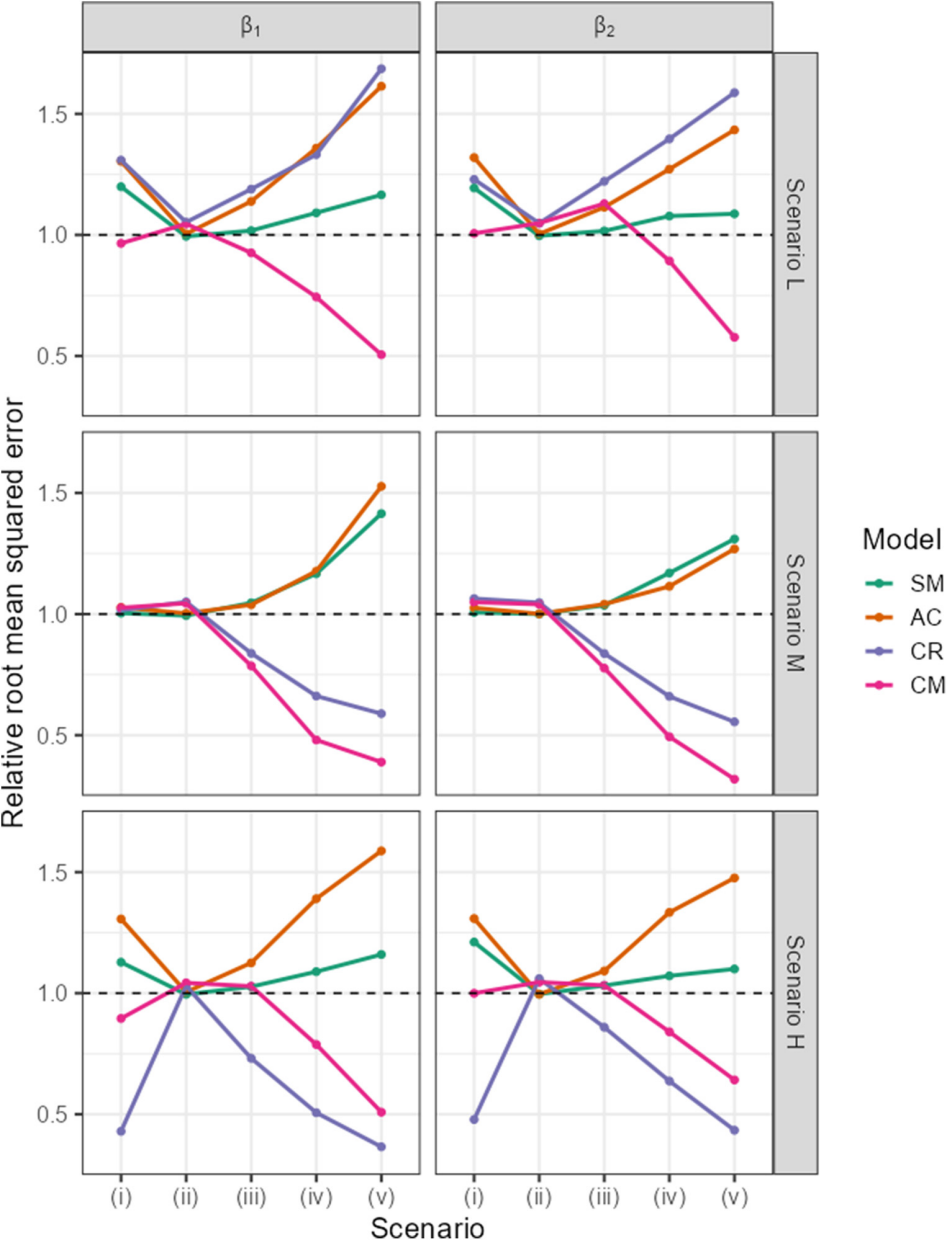
Patterns of relative root mean squared error for $\beta_{1}$ and $\beta_{2}$ from 1000 simulations comparing unweighted to weighted SM (stereotype model), AC (adjacent-category logit model), CR (continuation-ratio logit model), and CM (cumulative logit model), and across Scenarios L(i-v), M(i-v) with K=5 .

The simulation results of K=5 are presented in the main article (Tables 2 to 7 and Figure 1), and for K=3 are presented in the Supplemental Materials (Tables S7 to S12 and Figure S1).

**Table 2.** Percent (%) mean RB of the unweighted estimators under ODS: RB( ${\hat{\theta }}_{u}$ ); and mean absolute RB of the weighted estimators under ODS: RB( ${\hat{\theta }}_{w}$ ) from Scenarios L(i), L(iii), and L(v) with K=5 .

		Scenario L(i)			Scenario L(iii)			Scenario L(v)		
Model	θ	True	RB( ${\hat{\theta }}_{u}$ )	RB( ${\hat{\theta }}_{w}$ )	True	RB( ${\hat{\theta }}_{u}$ )	RB( ${\hat{\theta }}_{w}$ )	True	RB( ${\hat{\theta }}_{u}$ )	RB( ${\hat{\theta }}_{w}$ )
SM	$\alpha_{1}$	0.9	−173.3	−0.2	−1.6	−53.2	1.6	−3.4	−74.8	0.5
SM	$\alpha_{2}$	1.2	−128.3	0.9	−1.4	−70.9	1.1	−3.2	−82.5	0.7
SM	$\alpha_{3}$	1.3	−113.0	0.7	−1.3	−78.9	1.1	−3.2	−86.1	0.4
SM	$\alpha_{4}$	1.5	−103.0	0.5	−1.2	−85.8	1.0	−3.1	−88.5	0.4
SM	$\beta_{1}$	3.5	0.9	0.3	2.6	2.7	2.7	2.4	0.7	1.7
SM	$\phi_{2}\beta_{1}$	2.5	0.1	−0.9	1.7	2.9	3.3	1.7	1.5	3.3
SM	$\phi_{3}\beta_{1}$	1.9	0.5	−0.9	1.3	4.0	4.8	1.5	0.6	2.4
SM	$\phi_{4}\beta_{1}$	1.1	1.4	−1.5	0.9	4.5	6.2	1.3	1.0	3.3
SM	$\beta_{2}$	−1.7	1.6	0.8	−1.3	1.4	1.4	−1.2	0.9	0.7
SM	$\phi_{2}\beta_{2}$	−1.3	0.6	−0.7	−0.9	2.0	2.4	−0.9	1.5	2.2
SM	$\phi_{3}\beta_{2}$	−1.0	1.5	−0.2	−0.7	3.0	3.8	−0.7	0.6	1.5
SM	$\phi_{4}\beta_{2}$	−0.6	2.2	−0.8	−0.5	3.3	5.0	−0.6	0.6	2.1
AC	$\alpha_{1}$	1.5	−102.9	0.3	−1.2	−91.6	−0.3	−2.9	−94.8	0.1
AC	$\alpha_{2}$	−0.2	−35.5	1.1	−0.2	−1.7	−0.3	−0.2	19.8	2.0
AC	$\alpha_{3}$	−0.2	6.5	3.5	−0.2	17.7	−0.1	−0.2	33.8	5.9
AC	$\alpha_{4}$	−0.2	80.3	6.6	−0.1	132.3	6.4	−0.2	98.6	8.8
AC	$\beta_{1}$	0.8	1.5	1.7	0.6	−0.1	0.1	0.6	1.6	2.8
AC	$\beta_{2}$	−0.4	0.1	0.3	−0.3	0.2	0.1	−0.3	−0.3	0.7
CR	$\alpha_{1}$	2.7	−56.4	0.4	0.1	1171.7	−2.2	−1.7	−159.4	0.1
CR	$\alpha_{2}$	0.8	6.1	0.6	0.8	−4.7	−0.8	0.7	−6.6	−1.7
CR	$\alpha_{3}$	0.4	−11.9	0.2	0.4	−22.0	−2.9	0.3	−39.2	−6.6
CR	$\alpha_{4}$	−0.4	33.9	2.1	−0.4	40.1	5.4	−0.5	33.4	4.8
CR	$\beta_{1}$	1.3	−1.8	0.1	1.2	−1.6	1.5	1.3	−2.5	2.0
CR	$\beta_{2}$	−0.7	−1.1	1.0	−0.6	−1.6	0.9	−0.7	−2.7	1.3
CM	$\alpha_{1}$	−2.7	−57.0	0.2	0.0	−56435.3	−696.5	1.8	−153.1	0.6
CM	$\alpha_{2}$	−0.5	−85.3	0.0	0.6	−101.1	−1.5	2.2	−97.6	0.7
CM	$\alpha_{3}$	0.5	66.0	1.0	1.3	−32.4	−0.5	2.6	−65.9	0.6
CM	$\alpha_{4}$	1.6	20.8	0.7	2.1	−11.7	−0.1	3.3	−42.8	0.6
CM	$\beta_{1}$	−1.7	7.9	1.1	−1.7	−13.1	−0.3	−1.8	−33.6	2.0
CM	$\beta_{2}$	0.8	8.1	1.1	0.8	−12.0	1.3	0.9	−33.9	0.7

SM: stereotype model; AC: adjacent-category logit model; CR: continuation-ratio logit model; CM: cumulative logit model; RB: relative bias; ODS: outcome-dependent sampling.

**Table 3.** Percent (%) mean RB of the unweighted estimators under ODS: RB( ${\hat{\theta }}_{u}$ ); and percent mean RB of the weighted estimators under ODS: RB( ${\hat{\theta }}_{w}$ ) from Scenarios M(i), M(iii), and M(v) with K=5 .

		Scenario M(i)			Scenario M(iii)			Scenario M(v)		
Model	θ	True	RB( ${\hat{\theta }}_{u}$ )	RB( ${\hat{\theta }}_{w}$ )	True	RB( ${\hat{\theta }}_{u}$ )	RB( ${\hat{\theta }}_{w}$ )	True	RB( ${\hat{\theta }}_{u}$ )	RB( ${\hat{\theta }}_{w}$ )
SM	$\alpha_{1}$	−0.7	0.2	0.2	−0.8	−0.5	0.3	−1.0	1.2	3.7
SM	$\alpha_{2}$	−0.4	−29.7	−2.4	−0.6	−23.5	−0.2	−0.9	−17.3	1.9
SM	$\alpha_{3}$	−2.1	−90.2	−0.3	0.6	−138.4	0.6	2.3	−110.5	−0.5
SM	$\alpha_{4}$	−0.3	−49.5	−2.2	−0.2	−65.7	−1.1	−0.2	−92.1	0.8
SM	$\beta_{1}$	2.6	1.3	1.4	3.2	1.3	1.5	4.1	2.2	3.4
SM	$\phi_{2}\beta_{1}$	1.7	0.6	0.7	2.3	1.4	1.6	3.5	1.6	2.7
SM	$\phi_{3}\beta_{1}$	1.3	0.9	1.1	1.6	1.4	1.6	2.0	3.4	4.6
SM	$\phi_{4}\beta_{1}$	1.0	1.7	2.0	0.8	1.5	1.8	0.6	0.9	2.3
SM	$\beta_{2}$	−1.3	2.7	2.7	−1.6	2.3	2.5	−2.0	2.3	2.6
SM	$\phi_{2}\beta_{2}$	−0.8	2.0	1.9	−1.2	2.4	2.5	−1.7	1.6	1.8
SM	$\phi_{3}\beta_{2}$	−0.7	2.0	2.1	−0.8	2.1	2.2	−1.0	3.4	3.8
SM	$\phi_{4}\beta_{2}$	−0.5	3.2	3.2	−0.4	1.9	2.3	−0.3	0.7	1.6
AC	$\alpha_{1}$	−0.2	−62.3	−1.4	−0.2	−64.8	−0.0	−0.2	−85.9	−5.5
AC	$\alpha_{2}$	−1.9	−93.5	0.0	0.8	−119.9	−0.7	2.5	−107.5	0.1
AC	$\alpha_{3}$	1.6	−110.9	−0.0	−1.2	−79.3	0.7	−3.0	−88.3	0.1
AC	$\alpha_{4}$	−0.1	123.9	5.3	−0.2	77.2	7.1	−0.4	41.0	1.4
AC	$\beta_{1}$	0.6	1.0	1.1	0.8	1.5	1.9	1.1	−0.0	1.5
AC	$\beta_{2}$	−0.3	1.0	1.2	−0.4	0.3	0.6	−0.5	1.9	2.8
CR	$\alpha_{1}$	0.8	44.9	−0.3	1.4	−18.0	−0.1	2.5	−56.1	−0.2
CR	$\alpha_{2}$	0.5	76.2	−1.8	1.2	−39.9	−0.2	2.5	−74.2	−0.3
CR	$\alpha_{3}$	2.2	−82.7	−0.4	−0.6	−138.3	1.0	−2.5	−100.7	0.7
CR	$\alpha_{4}$	−0.3	20.3	5.5	−0.4	45.7	2.6	−0.6	41.7	3.6
CR	$\beta_{1}$	1.3	−11.8	2.0	1.3	6.6	0.5	1.7	15.7	2.3
CR	$\beta_{2}$	−0.6	−12.1	1.4	−0.7	6.2	0.0	−0.8	16.0	1.6
CM	$\alpha_{1}$	−0.8	46.8	−0.2	−1.3	−17.9	−0.0	−2.5	−54.9	−0.2
CM	$\alpha_{2}$	0.3	−118.8	2.2	−0.4	−101.6	−0.7	−1.7	−103.3	−0.4
CM	$\alpha_{3}$	0.5	63.0	1.7	1.3	−28.5	0.8	2.7	−59.9	0.9
CM	$\alpha_{4}$	1.6	17.3	1.0	2.2	−7.6	0.6	3.4	−34.1	0.8
CM	$\beta_{1}$	−1.6	−10.0	1.3	−1.7	8.5	1.2	−1.9	22.6	2.5
CM	$\beta_{2}$	0.8	−10.9	0.3	0.8	8.4	1.0	1.0	25.1	2.6

SM: stereotype model; AC: adjacent-category logit model; CR: continuation-ratio logit model; CM: cumulative logit model; RB: relative bias; ODS: outcome-dependent sampling.

**Table 4.** Percent (%) mean RB of the unweighted estimators under ODS: RB( ${\hat{\theta }}_{u}$ ); and percent mean RB of the weighted estimators under ODS: RB( ${\hat{\theta }}_{w}$ ) from Scenarios H(i), H(iii), and H(v) with K=5 .

		Scenario H(i)			Scenario H(iii)			Scenario H(v)		
Model	θ	True	RB( ${\hat{\theta }}_{u}$ )	RB( ${\hat{\theta }}_{w}$ )	True	RB( ${\hat{\theta }}_{u}$ )	RB( ${\hat{\theta }}_{w}$ )	True	RB( ${\hat{\theta }}_{u}$ )	RB( ${\hat{\theta }}_{w}$ )
SM	$\alpha_{1}$	−2.7	−59.5	0.6	0.3	−267.9	−9.2	2.2	−114.1	0.6
SM	$\alpha_{2}$	−0.6	−8.2	1.7	−0.4	−38.8	2.1	−0.3	−83.1	−4.0
SM	$\alpha_{3}$	−0.4	−30.5	0.7	−0.3	−48.7	1.9	−0.2	−89.5	−4.6
SM	$\alpha_{4}$	−0.3	−45.4	2.5	−0.2	−60.5	1.1	−0.2	−95.1	−5.4
SM	$\beta_{1}$	3.5	1.7	1.2	2.6	2.4	2.5	2.4	0.3	0.9
SM	$\phi_{2}\beta_{1}$	2.4	1.0	1.7	1.6	2.2	1.3	1.1	1.1	0.0
SM	$\phi_{3}\beta_{1}$	1.6	0.9	1.5	1.3	2.0	1.1	0.9	1.1	0.2
SM	$\phi_{4}\beta_{1}$	1.0	3.3	4.1	0.8	2.2	1.4	0.6	2.3	1.5
SM	$\beta_{2}$	−1.7	2.4	1.3	−1.3	0.4	0.7	−1.2	2.9	3.3
SM	$\phi_{2}\beta_{2}$	−1.2	1.6	1.7	−0.8	0.5	−0.2	−0.6	3.7	2.4
SM	$\phi_{3}\beta_{2}$	−0.8	1.4	1.5	−0.6	−0.2	−0.8	−0.5	4.3	3.0
SM	$\phi_{4}\beta_{2}$	−0.5	3.9	4.2	−0.4	0.8	0.0	−0.3	4.6	3.7
AC	$\alpha_{1}$	−0.2	−55.2	−3.2	−0.2	−71.9	−2.0	−0.1	−102.4	−3.2
AC	$\alpha_{2}$	−0.2	−4.4	−1.5	−0.1	−26.1	0.4	−0.1	−58.3	−1.6
AC	$\alpha_{3}$	−0.2	36.4	2.9	−0.2	−3.0	1.5	−0.1	−20.2	4.6
AC	$\alpha_{4}$	−1.9	−81.1	0.3	0.9	−124.6	−1.0	2.6	−106.1	−0.3
AC	$\beta_{1}$	0.8	0.6	0.7	0.6	0.8	1.0	0.6	0.6	2.9
AC	$\beta_{2}$	−0.4	1.9	2.1	−0.3	1.1	0.9	−0.3	2.3	3.6
CR	$\alpha_{1}$	0.8	27.0	0.2	1.4	−10.3	−0.0	2.5	−46.5	−0.4
CR	$\alpha_{2}$	0.5	35.1	−0.8	1.3	−27.9	−0.4	2.5	−60.0	−0.5
CR	$\alpha_{3}$	−0.2	−152.0	4.5	1.0	−55.7	−0.7	2.5	−77.0	−0.7
CR	$\alpha_{4}$	−2.2	−64.4	0.7	0.7	−147.9	−2.1	2.5	−107.1	−0.8
CR	$\beta_{1}$	1.3	22.9	1.0	1.3	−20.9	1.0	1.6	−44.1	2.3
CR	$\beta_{2}$	−0.7	23.1	1.4	−0.6	−21.1	0.7	−0.8	−43.8	1.4
CM	$\alpha_{1}$	−0.7	33.8	−0.6	−1.3	−11.0	0.1	−2.5	−46.0	−0.1
CM	$\alpha_{2}$	0.3	−75.3	3.2	−0.4	−68.6	−0.8	−1.8	−81.7	−0.4
CM	$\alpha_{3}$	1.4	−27.8	1.3	0.2	249.1	2.9	−1.3	−139.8	−0.8
CM	$\alpha_{4}$	3.5	−41.3	0.7	0.8	114.5	1.1	−1.0	−260.1	−1.2
CM	$\beta_{1}$	−1.7	8.9	1.8	−1.7	−12.2	0.3	−1.8	−33.7	1.9
CM	$\beta_{2}$	0.8	7.3	0.6	0.8	−13.1	0.0	0.9	−32.6	2.2

SM: stereotype model; AC: adjacent-category logit model; CR: continuation-ratio logit model; CM: cumulative logit model; RB: relative bias; ODS: outcome-dependent sampling.

**Table 5.** Percent (%) RE of model-based standard errors and percent RE of design-based standard errors of weighted models under ODS from Scenarios L(i), L(iii), and L(v) with K=5 .

		Scenario L(i)		Scenario L(iii)		Scenario L(v)	
Model	θ	$\text{RE}({\hat{\theta }}_{u})$	$\text{RE}({\hat{\theta }}_{w})$	$\text{RE}({\hat{\theta }}_{u})$	$\text{RE}({\hat{\theta }}_{w})$	$\text{RE}({\hat{\theta }}_{u})$	$\text{RE}({\hat{\theta }}_{w})$
SM	$\alpha_{1}$	8.1	0.9	5.4	−0.3	2.0	−4.7
SM	$\alpha_{2}$	14.2	−2.2	17.1	0.6	5.9	−6.3
SM	$\alpha_{3}$	32.7	1.2	25.4	−0.8	11.9	−4.2
SM	$\alpha_{4}$	69.8	−4.5	38.7	−3.7	16.2	−6.1
SM	$\beta_{1}$	2.1	3.0	−3.4	−0.6	−8.4	−6.3
SM	$\phi_{2}\beta_{1}$	−0.7	1.1	−3.3	−1.0	−6.5	−5.0
SM	$\phi_{3}\beta_{1}$	0.3	2.3	−3.7	−0.9	−7.0	−3.9
SM	$\phi_{4}\beta_{1}$	−0.2	2.6	−7.3	−4.4	−6.4	−3.3
SM	$\beta_{2}$	−3.8	0.2	−3.8	−0.1	−2.1	1.6
SM	$\phi_{2}\beta_{2}$	−1.9	2.3	−7.0	−3.9	0.2	2.9
SM	$\phi_{3}\beta_{2}$	−5.5	−0.7	−6.7	−3.8	−1.8	2.2
SM	$\phi_{4}\beta_{2}$	−1.8	1.3	−6.9	−3.8	−0.8	2.1
AC	$\alpha_{1}$	129.2	−12.0	138.8	−10.1	134.6	−15.1
AC	$\alpha_{2}$	126.5	−8.4	132.0	−11.6	121.4	−21.7
AC	$\alpha_{3}$	111.6	−6.2	118.6	−7.8	104.7	−18.0
AC	$\alpha_{4}$	92.0	−4.9	103.6	−9.1	89.7	−15.5
AC	$\beta_{1}$	−3.2	−2.3	0.4	−0.5	−1.3	−5.6
AC	$\beta_{2}$	−3.0	−2.3	−1.5	−1.3	−0.6	−1.3
CR	$\alpha_{1}$	35.5	−5.7	40.6	1.0	33.6	−1.4
CR	$\alpha_{2}$	35.4	−3.8	38.3	0.3	30.8	−4.5
CR	$\alpha_{3}$	34.6	−4.8	35.8	0.5	27.8	−3.5
CR	$\alpha_{4}$	35.7	−5.0	35.9	−0.6	28.3	−5.1
CR	$\beta_{1}$	0.1	−3.7	1.3	0.5	0.1	−1.6
CR	$\beta_{2}$	−1.4	−1.3	2.3	0.2	1.2	1.2
CM	$\alpha_{1}$	24.0	−3.7	20.2	−2.2	15.4	−0.8
CM	$\alpha_{2}$	15.6	−1.7	11.7	−2.4	7.0	−0.6
CM	$\alpha_{3}$	13.7	−1.7	10.0	−2.0	5.8	−0.9
CM	$\alpha_{4}$	17.1	−1.8	13.7	−2.2	9.8	−1.4
CM	$\beta_{1}$	−0.3	−1.2	−0.9	1.0	−3.2	0.0
CM	$\beta_{2}$	0.5	−1.1	1.2	−0.4	0.4	0.5

ODS: outcome-dependent sampling; SM: stereotype model; AC: adjacent-category logit model; CR: continuation-ratio logit model; CM: cumulative logit model; RE: relative error.

**Table 6.** Percent (%) RE of model-based standard errors and percent RE of design-based standard errors of weighted models under ODS from Scenarios M(i), M(iii), and M(v) with K=5 .

		Scenario M(i)		Scenario M(iii)		Scenario M(v)	
Model	θ	$\text{RE}({\hat{\theta }}_{u})$	$\text{RE}({\hat{\theta }}_{w})$	$\text{RE}({\hat{\theta }}_{u})$	$\text{RE}({\hat{\theta }}_{w})$	$\text{RE}({\hat{\theta }}_{u})$	$\text{RE}({\hat{\theta }}_{w})$
SM	$\alpha_{1}$	2.4	−3.1	8.4	2.0	14.3	5.6
SM	$\alpha_{2}$	18.4	−0.4	15.0	−0.3	13.0	3.3
SM	$\alpha_{3}$	26.2	−6.0	30.8	−1.5	41.0	2.2
SM	$\alpha_{4}$	47.3	−2.0	89.1	−1.0	195.4	−16.6
SM	$\beta_{1}$	−7.3	−4.6	−2.7	−0.1	1.1	3.3
SM	$\phi_{2}\beta_{1}$	−4.4	−1.6	−3.6	−1.4	−3.1	1.2
SM	$\phi_{3}\beta_{1}$	−4.8	−2.1	−5.7	−3.9	0.0	−0.0
SM	$\phi_{4}\beta_{1}$	−1.6	1.0	−3.2	−1.3	−3.8	−1.9
SM	$\beta_{2}$	−1.6	1.3	−0.2	3.3	−3.5	−1.4
SM	$\phi_{2}\beta_{2}$	−2.5	0.5	−1.8	1.2	−1.8	−0.3
SM	$\phi_{3}\beta_{2}$	−0.2	2.8	−3.0	−1.1	−1.2	−2.2
SM	$\phi_{4}\beta_{2}$	−0.7	2.1	−0.6	1.6	−2.6	−1.5
AC	$\alpha_{1}$	150.5	−6.8	136.4	−11.3	112.1	−18.2
AC	$\alpha_{2}$	144.6	−17.3	129.0	−10.1	114.8	−8.0
AC	$\alpha_{3}$	131.2	−16.6	110.5	−8.4	100.3	−9.1
AC	$\alpha_{4}$	114.6	−2.6	90.2	−10.3	80.3	−7.5
AC	$\beta_{1}$	2.5	2.4	−3.0	−2.7	3.7	1.9
AC	$\beta_{2}$	1.9	1.7	−1.0	−1.2	−0.6	1.7
CR	$\alpha_{1}$	37.0	−1.7	35.4	−3.3	24.1	−10.5
CR	$\alpha_{2}$	35.6	−2.5	33.4	−2.8	22.0	−11.0
CR	$\alpha_{3}$	35.9	−5.6	30.6	−2.9	15.7	−8.3
CR	$\alpha_{4}$	37.4	−1.1	30.9	−4.3	18.1	−8.2
CR	$\beta_{1}$	0.3	−1.8	−0.9	−2.1	−4.6	−4.6
CR	$\beta_{2}$	1.5	−0.3	0.7	0.5	−0.1	−1.0
CM	$\alpha_{1}$	19.7	−3.7	20.0	−3.3	15.9	−1.5
CM	$\alpha_{2}$	11.3	−3.4	11.6	−3.6	8.8	−0.3
CM	$\alpha_{3}$	10.1	−3.2	9.2	−4.1	7.0	−2.9
CM	$\alpha_{4}$	14.4	−2.8	11.8	−4.4	9.8	−3.3
CM	$\beta_{1}$	−0.8	−1.4	−2.7	−4.2	0.7	−2.2
CM	$\beta_{2}$	0.2	−0.7	−2.8	−2.8	1.4	1.8

ODS: outcome-dependent sampling; SM: stereotype model; AC: adjacent-category logit model; CR: continuation-ratio logit model; CM: cumulative logit model; RE: relative error.

**Table 7.** Percent (%) RE of model-based standard errors and percent RE of design-based standard errors of weighted models under ODS from Scenarios H(i), H(iii), and H(v) with K=5 .

		Scenario H(i)		Scenario H(iii)		Scenario H(v)	
Model	θ	$\text{RE}({\hat{\theta }}_{u})$	$\text{RE}({\hat{\theta }}_{w})$	$\text{RE}({\hat{\theta }}_{u})$	$\text{RE}({\hat{\theta }}_{w})$	$\text{RE}({\hat{\theta }}_{u})$	$\text{RE}({\hat{\theta }}_{w})$
SM	$\alpha_{1}$	6.6	2.2	4.3	−2.0	5.5	−0.7
SM	$\alpha_{2}$	16.7	1.1	21.4	−0.0	32.1	−6.0
SM	$\alpha_{3}$	34.1	0.4	30.2	−2.5	42.9	−5.3
SM	$\alpha_{4}$	68.1	1.3	61.7	−0.1	67.2	−8.0
SM	$\beta_{1}$	−1.6	2.6	−6.3	−3.8	−4.3	−1.7
SM	$\phi_{2}\beta_{1}$	−1.0	2.0	−5.8	−2.7	−5.0	−0.9
SM	$\phi_{3}\beta_{1}$	−1.8	1.3	−6.7	−2.8	−2.4	1.8
SM	$\phi_{4}\beta_{1}$	−2.6	0.3	−3.3	−0.1	−2.1	2.0
SM	$\beta_{2}$	0.5	3.7	−3.6	−0.6	−4.7	−1.7
SM	$\phi_{2}\beta_{2}$	−0.8	1.6	−7.1	−4.0	−5.3	−1.8
SM	$\phi_{3}\beta_{2}$	−1.4	0.1	−4.1	−1.4	−6.5	−2.3
SM	$\phi_{4}\beta_{2}$	−4.0	−2.2	−4.8	−1.7	−3.4	0.7
AC	$\alpha_{1}$	143.7	−5.4	149.2	−8.0	134.2	−18.0
AC	$\alpha_{2}$	133.4	−4.7	147.8	−8.5	135.5	−18.6
AC	$\alpha_{3}$	110.9	−4.6	134.7	−8.7	128.8	−19.5
AC	$\alpha_{4}$	88.9	−11.5	115.4	−3.9	114.7	−9.9
AC	$\beta_{1}$	−2.9	−2.3	−0.4	−0.4	−0.7	−3.6
AC	$\beta_{2}$	−1.9	−0.9	−5.2	−4.0	−1.2	−2.9
CR	$\alpha_{1}$	35.5	0.3	40.5	1.6	42.4	−1.0
CR	$\alpha_{2}$	33.0	−0.2	40.6	1.3	44.6	−0.7
CR	$\alpha_{3}$	28.5	−2.2	41.5	2.3	47.5	−1.4
CR	$\alpha_{4}$	26.3	−5.1	45.3	0.5	54.1	−1.3
CR	$\beta_{1}$	−0.9	−1.9	1.7	1.0	5.7	−1.3
CR	$\beta_{2}$	−2.1	−1.0	−2.0	−2.0	0.7	−0.5
CM	$\alpha_{1}$	26.5	2.6	25.2	−0.9	20.2	−1.4
CM	$\alpha_{2}$	17.4	3.2	16.3	−0.7	12.9	−0.4
CM	$\alpha_{3}$	14.9	3.2	14.6	−0.5	12.6	0.0
CM	$\alpha_{4}$	17.6	2.8	18.8	−0.9	18.1	−0.0
CM	$\beta_{1}$	2.7	3.3	0.3	−1.0	−2.8	−1.4
CM	$\beta_{2}$	1.9	1.6	0.6	1.1	−1.2	−2.4

ODS: outcome-dependent sampling; SM: stereotype model; AC: adjacent-category logit model; CR: continuation-ratio logit model; CM: cumulative logit model; RE: relative error.

Table 2 shows the percent mean relative biases, RB( ${\hat{\theta }}_{u}$ ) and RB( ${\hat{\theta }}_{w}$ ), from simulation Scenarios L(i), L(iii), and L(v) for K=5 . We also present θ for each scenario because they are different for each model. As expected, the unweighted SM produced log odds estimates, $({\hat{\phi }}_{k}\hat{\beta }{)}_{u}$ , with low RB for all k=1,2,3,4 (note that $\phi_{1}=1$ for identifiability), but produced intercept estimates, ${\hat{\alpha }}_{u}$ , with considerably larger RB compared to the log odds estimates. The weighted SM produced $({\hat{\phi }}_{k}\hat{\beta }{)}_{w}$ with RB in a range similar to $({\hat{\phi }}_{k}\hat{\beta }{)}_{u}$ . For some log odds estimates in certain scenarios, the RB in $({\hat{\phi }}_{k}\hat{\beta }{)}_{w}$ was larger. For example, RB for $({\hat{\phi }}_{4}{\hat{\beta }}_{1}{)}_{u}$ in Scenario L(iii) was 4.51% in unweighted SM, but RB for $({\hat{\phi }}_{4}{\hat{\beta }}_{1}{)}_{w}$ was 6.23%. On the other hand, weighted SM produced ${\hat{\alpha }}_{w}$ with negligible RB.

Also as expected, unweighted AC produced ${\hat{\beta }}_{u}$ with negligible RB but produced ${\hat{\alpha }}_{u}$ with extremely large RB. Similarly to weighted SM, weighted AC produced ${\hat{\beta }}_{w}$ with a range of RB similar to that of their unweighted counterparts. The RB for ${\hat{\alpha }}_{w}$ was negligible except for a few estimates where the true values were close to 0. In this scenario, where $f_{k}$ is approximately equal for k=2,…,K , unweighted CR produced ${\hat{\beta }}_{u}$ with negligible RB (see Appendix II). The weighted CR also produced ${\hat{\beta }}_{w}$ with small RB. Similarly to the two models above, RB for ${\hat{\alpha }}_{u}$ was extremely large, but RB for ${\hat{\alpha }}_{w}$ was much smaller. The unweighted CM produced ${\hat{\beta }}_{u}$ with the largest RB among all models. The largest RB was observed in the most extreme scenario, L(v). RB for ${\hat{\alpha }}_{u}$ was also extremely large. The weighted CM produced ${\hat{\beta }}_{w}$ and ${\hat{\alpha }}_{w}$ with minimal RB, clearly demonstrating the advantage of the weighted model.

Table 3 shows the equivalent for Scenarios M(i), M(iii), and M(v). We observed a similar pattern where unweighted SM and AC produced unbiased or low-biased log odds estimates but produced biased intercept estimates, and weighted models produced similarly unbiased or low-biased log odds estimates and unbiased intercept estimates. On the other hand, the RB of the log odds estimates in the unweighted CR was much larger compared to those in the L scenario. The unweighted CM also produced log odds estimates with similarly large RB, which was also observed in the L scenarios. In both models, the weighted log odds estimates had negligible RB. Table 4 shows the equivalent for Scenarios H(i), H(iii), and H(v). Similar patterns were observed in all models. However, compared to the other scenarios, the unweighted CR produced log odds estimates with the largest RB in this scenario.

Table 5 shows $\text{RE}({\hat{\theta }}_{u})$ and $\text{RE}({\hat{\theta }}_{w})$ for Scenarios L(i), L(iii), and L(v). For SM, most $\text{RE}({\hat{\theta }}_{u})$ of the log odds estimates were less than 0 in all scenarios, indicating an undercoverage of model-based standard errors. The $\text{RE}({\hat{\theta }}_{w})$ of the log odds estimates and the intercepts were closer to 0, indicating a better coverage of the design-based standard errors. For AC, $\text{RE}({\hat{\theta }}_{w})$ of the log odds estimates were also close to 0 indicating a good coverage of the design-based standard errors. However, $\text{RE}({\hat{\theta }}_{w})$ of the intercepts were negative and large. For CR, $\text{RE}({\hat{\theta }}_{w})$ of the log odds estimates were small, ranging between −3.2% and 1.8%, but $\text{RE}({\hat{\theta }}_{w})$ of the intercept had a larger range between −15.7% and 1.6%. For CM, $\text{RE}({\hat{\theta }}_{w})$ of the log odds estimates and intercepts were both small. Similar patterns were observed for Scenarios M(i), M(iii), and M(v) shown in Table 6. For scenarios H(i), H(iii), and H(v), $\text{RE}({\hat{\theta }}_{u})$ and $\text{RE}({\hat{\theta }}_{w})$ were similar for the log odds estimates in all models (Table 7). In general, $\text{RE}({\hat{\theta }}_{w})$ of the log odds estimates were larger compared to those for Scenarios L and M.

Figure 1 shows the RRMSE of ${\hat{\beta }}_{w}$ to ${\hat{\beta }}_{u}$ from each model in all scenarios when K=5 . The results of the K=3 scenario were similar and are shown in the Supplemental Material. As expected, the RRMSE was close to one for all models when the proportions of the outcome categories were evenly distributed in the cohort (Scenarios L(ii), M(ii), and H(ii) are equivalent). For SM and AC, RRMSE was above one or close to one in both parameters in all scenarios, indicating that the weighted estimates were less efficient than the unweighted estimates. In Scenarios L and H, the RRMSE for AC was always larger than SM. In Scenario M, the RRMSE for SM and AC was almost identical. However, for CR and CM, the RRMSE was below one in many of the scenarios. For Scenarios M and H, the RRMSE patterns were similar between CR and CM for both parameters. For Scenario L, the RRMSE was above one for CR (similar to AC), but below one (similar to Scenarios M and H) for CM. Generally, low biased estimates (SM and AC in all scenarios, and CR in Scenario L) had RRMSE greater than one, and highly biased estimates had RRMSE less than one (CR in Scenarios M and H, and CM in all scenarios).

## 5. Case study

We illustrated the use of different models for ordinal outcomes with data from a study of knee osteoarthritis (OA), a chronic debilitating disease that affects an estimated 654.1 million people aged 40 and older worldwide.^27^ Currently, there is no cure for OA and existing treatments may be inadequate for a significant portion of participants who have to cope with long-lasting pain. Thus, identifying risk factors and biomarkers is necessary to develop preventive strategies and treatment options in the early stages of the disease. In knee OA, the KL radiographic grade is commonly used to determine the severity of the disease. The KL grade ranges from 0 to 4, where grade 0 indicates the absence of OAs and grade 4 indicates the most severe OA condition.^28^

The OA initiative (OAI) is a multicenter cohort study started in 2002 designed to identify risk factors for knee OA. A total of 4796 participants aged 45–79 years were recruited into one of three subcohorts: progression ( ≈ 29%), incidence ( ≈ 68%) and control ( ≈ 3%).^29^ In summary, the progression subcohort corresponds to participants with symptomatic radiographic knee OA. The incidence subcohort corresponds to participants considered to have a higher risk of developing symptomatic radiographic knee OA based on the clinical and demographic factors such as weight, family history of knee replacement, previous knee injuries, or surgeries, and hand OA. The control subcohort corresponds to a reduced number of participants without symptomatic radiographic knee OA or risk factors. Major exclusion criteria include inflammatory arthritis, contraindication to 3T MRI, and bilateral end-stage knee OA. More details of the study are available on the OAI website (https://nda.nih.gov/oai/study-details).

As part of the OAI, a comprehensive questionnaire was collected along with imaging and biospecimen data from consenting participants with a maximum of seven visits (yearly from years 0 to 4 and every 2 years thereafter) over a span of ∼ 10 years. At baseline, the study consisted of 58% women, 51% over 60 years of age, and 37% with a body mass index (BMI) > 30. Recognizing the important role of genetics in OA,^30,31^ researchers from OAI genotyped participants as part of a large genome-wide association study.^32^ Although no variants met the stringent genome-wide significant threshold, this landmark study allowed investigators to observe a nominal association and validate previous findings. For example, the FTO gene, which is involved in body weight regulation, has previously been reported to be linked to an increased risk of OA.^33,34^

We focused on modeling the association between the five-category KL grade ( $Y_{i}$ ) obtained at baseline and the predicted expression of gene FTO ( $X_{i}$ ), adjusting for baseline age ( $Z_{1i}$ ), sex ( $Z_{2i}$ ), BMI ( $Z_{3i}$ ), and cohort membership ( $Z_{4i}$ ). We show the distribution of the variables of interest in Table 8. Note that the distribution of KL grade is most similar to Scenario H(v) in the simulation study (Table 1). In this case study, we considered the cohort of 3855 participants in the OAI to be the “full cohort.” Note that the cohort itself is not an ODS of the population of knee OA patients and because the outcome sampling probability is unknown, an inference about the population cannot be made. As a hypothetical scenario, we collect ( $Y_{i},Z_{1i},\ldots ,Z_{4i}$ ) on all 3855 participants, but only have resources to ascertain $X_{i}$ from an ODS sample of 150 participants in each KL grade category. Although collecting genotyping data using chip arrays has become relatively affordable for most studies, there are instances where resources may be preferred to be used elsewhere, for example, to collect information from other technologies. In this context, $f_{k}$ for each k=1,…,5 is known. We then fit the four models (SM, AC, CR, and CM) to the full cohort and to the ODS sample, with and without weights. Our interest was to compare the estimated log odds of FTO on KL grade from the unweighted ( ${\hat{\beta }}_{u}$ ) and weighted ( ${\hat{\beta }}_{w}$ ) models to the full cohort ( ${\hat{\beta }}_{F}$ ) model. To quantify the variability introduced by the sampling scheme, we repeated the drawing 200 times and aggregated the results. In Table 9, we reported the mean relative bias and RRMSE as defined in Section 4 as well as relative efficiency, $\hat{\text{SE}}({\hat{\beta }}_{u|w})/\hat{\text{SE}}({\hat{\beta }}_{F})$ , where $\hat{\text{SE}}({\hat{\beta }}_{u|w})$ is the mean of the standard error estimates obtained under ODS ( n=750 ) across 200 replicates and $\hat{\text{SE}}({\hat{\beta }}_{F})$ is the standard error estimate obtained from the full data ( N=3855 ).

**Table 8.** Distribution of variables of interest separated by full, progression, and incidence cohorts from the longitudinal study of knee osteoarthritis. Categorical variables are reported by their counts (percentages) whereas continuous variables are reported by their mean (sd) and median (range), as indicated.

	Full cohort	Progression cohort	Incidence cohort
Number of participants	3855	1111	2744
Maximum KL grade^*^			
0	1031 (27)	64 (6)	967 (35)
1	596 (15)	81 (7)	515 (19)
2	1200 (31)	368 (33)	832 (30)
3	770 (20)	411 (37)	359 (13)
4	258 (7)	187 (17)	71 (3)
Sex			
Female	2211 (57)	604 (54)	1607 (59)
Male	1644 (43)	507 (46)	1137 (41)
FTO			
Mean (sd)	0.1 (0.1)	0.1 (0.1)	0.1 (0.1)
Median (min, max)	0.2 (−0.6, 0.3)	0.2 (−0.4, 0.3)	0.2 (−0.6, 0.3)
BMI (baseline)			
Mean (sd)	28.7 (4.8)	30.1 (4.8)	28.2 (4.7)
Median (min, max)	28.4 (17.2, 47.7)	29.6 (18.2, 47.7)	27.9 (17.2, 46.8)
Age (baseline)			
Mean (sd)	61.4 (9.1)	61.3 (9.1)	61.4 (9.2)
Median (min, max)	61 (45, 79)	61 (45, 79)	61 (45, 79)

^*^ KL grade: Kellgren-Lawrence (KL) radiographic grade.

**Table 9.** Percent RB, Rel eff, and RRMSE of the log odds of FTO predicted expression on the KL grade estimated with unweighted and weighted models across 200 ODS samples compared to full cohort from the study of knee osteoarthritis.

	Unweighted		Weighted		
Model	RB (%)	Rel eff	RB (%)	Rel eff	RRMSE
SM	7.6	2.208	8.2	2.507	1.154
AC	1.6	2.100	7.9	2.437	1.221
CR	19.1	2.220	10.1	2.306	1.053
CM	13.5	2.253	9.5	2.452	1.141

RB: relative bias; Rel eff: relative efficiency; RRMSE: relative root mean square; FTO: Fat mass and obesity-associated; ODS: outcome-dependent sampling; SM: stereotype model; AC: adjacent-category logit model; CR: continuation-ratio logit model; CM: cumulative logit model.

Consistent with the results of our simulation study, the results of the case study showed that the unweighted SM and AC models produced less biased estimates compared to the unweighted CM and CR models. With weights, these relative biases were more comparable across all models, with slightly smaller bias in the weighted SM and AC models. The relative efficiencies of the weighted estimates were higher than those of the unweighted estimates for all models. Lastly, the RRMSE values were 1.154 for SM, 1.221 for AC, 1.141 for CM, and 1.053 for CR, suggesting that the weighted estimates were less efficient than the unweighted ones (Table 9).

The interpretation of these log odds estimates depends on the model. Table 10 shows the parameter estimates from the four ordinal regression models fitted to the full data and one ODS data. For example, based on weighted SM, the log odds ratio (SE) of observing $Y_{i}=5$ versus $Y_{i}=1$ (KL 4 vs. 0) per unit increase in FTO predicted expression was 1.490 (1.039). And based on the weighted AC, the log odds of any two adjacent KL grades for a one unit increase in FTO was 0.421 (0.268).

**Table 10.** Parameter estimates and SE estimates from the full data and ODS data with unweighted and weighted models applied to the study of knee osteoarthritis.

		Full		ODS (unweighted)		ODS (weighted)	
Model	θ	${\hat{\theta }}_{F}$	$\hat{\text{SE}}({\hat{\theta }}_{F}$ )	${\hat{\theta }}_{u}$	$\hat{\text{SE}}({\hat{\theta }}_{u}$ )	${\hat{\theta }}_{w}$	$\hat{\text{SE}}({\hat{\theta }}_{w}$ )
SM	$\alpha_{1}$	−9.782	0.657	−6.988	1.321	−9.163	1.407
SM	$\alpha_{2}$	−7.105	0.526	−4.951	1.038	−5.970	1.089
SM	$\alpha_{3}$	−3.985	0.339	−3.167	0.741	−3.554	0.793
SM	$\alpha_{4}$	−2.565	0.279	−1.026	0.508	−1.739	0.549
SM	$\phi_{2}$	0.841	0.033	0.761	0.053	0.774	0.053
SM	$\phi_{3}$	0.545	0.027	0.521	0.059	0.536	0.064
SM	$\phi_{4}$	0.279	0.032	0.182	0.075	0.185	0.075
SM	FTO	1.182	0.408	1.582	0.883	1.490	1.039
SM	Male versus female	0.221	0.118	0.491	0.255	0.227	0.284
SM	BMI	0.156	0.013	0.128	0.028	0.156	0.030
SM	Age	0.099	0.007	0.090	0.015	0.091	0.016
SM	Inc versus Prog	−3.625	0.185	−3.959	0.382	−3.822	0.405
AC	$\alpha_{1}$	−2.448	0.160	−1.453	0.337	−2.249	0.338
AC	$\alpha_{2}$	−1.396	0.166	−1.628	0.350	−1.184	0.354
AC	$\alpha_{3}$	−2.805	0.170	−1.886	0.360	−2.588	0.366
AC	$\alpha_{4}$	−3.745	0.185	−2.188	0.369	−3.537	0.377
AC	FTO	0.317	0.109	0.432	0.226	0.421	0.268
AC	Male versus female	0.069	0.031	0.134	0.064	0.070	0.071
AC	BMI	0.041	0.003	0.032	0.007	0.040	0.008
AC	Age	0.026	0.002	0.023	0.004	0.024	0.004
AC	Inc versus Prog	−0.966	0.040	−1.022	0.084	−0.993	0.095
CR	$\alpha_{1}$	−2.266	0.244	−1.251	0.544	−1.910	0.523
CR	$\alpha_{2}$	−2.119	0.248	−1.733	0.553	−1.741	0.536
CR	$\alpha_{3}$	−3.941	0.255	−2.447	0.563	−3.575	0.553
CR	$\alpha_{4}$	−5.329	0.269	−3.529	0.574	−4.937	0.567
CR	FTO	0.428	0.172	0.652	0.377	0.600	0.399
CR	Male versus female	0.188	0.048	0.311	0.107	0.182	0.107
CR	BMI	0.060	0.005	0.046	0.012	0.057	0.011
CR	Age	0.043	0.003	0.040	0.006	0.039	0.006
CR	Inc versus Prog	−1.473	0.059	−1.683	0.131	−1.511	0.122
CM	$\alpha_{1}$	3.011	0.301	1.885	0.681	2.492	0.687
CM	$\alpha_{2}$	3.850	0.304	3.095	0.689	3.322	0.698
CM	$\alpha_{3}$	5.540	0.310	4.209	0.696	5.002	0.711
CM	$\alpha_{4}$	7.447	0.319	5.528	0.704	6.900	0.721
CM	FTO	−0.526	0.213	−0.858	0.474	−0.761	0.529
CM	Male versus female	−0.144	0.060	−0.293	0.134	−0.148	0.141
CM	BMI	−0.080	0.006	−0.069	0.015	−0.078	0.015
CM	Age	−0.052	0.003	−0.048	0.008	−0.046	0.008
CM	Inc versus Prog	1.892	0.072	2.225	0.163	1.938	0.158

Inc: incidence; Prog: progression; SE: standard error; Rel eff: relative efficiency; RRMSE: relative root mean square; FTO: Fat mass and obesity-associated; BMI: body mass index; ODS: outcome-dependent sampling; SM: stereotype model; AC: adjacent-category logit model; CR: continuation-ratio logit model; CM: cumulative logit model.

## 6. Discussion

We investigated the finite sample properties of unweighted and weighted ordinal regression models under ODS from a cohort with various distributions of the ordinal outcome categories. Through simulation based on synthetic data and a cohort study investigating knee OA, we compared the bias and efficiency of four representative ordinal regression models: the SM; AM; CR; and CM, with and without inverse sampling weights. As shown in our derivation in Appendix I and pointed out by others,^7,13,35^ the log odds estimates from the unweighted SM and AM are consistent under ODS, but their finite sample properties, including bias and efficiency, of unweighted and weighted SMs had never been investigated previously. Through our numerical investigations, we observed that the unweighted SM and the unweighted adjacent-category logit model produce consistent log odds estimates while the unweighted cumulative logit model while the unweighted continuation-ratio logit model produces biased log odds estimates under various scenarios. However, under ODS, the sampling probabilities from each ordinal outcome category are usually known. In that case, including the inverse of the sampling probability as weights can improve the bias of the intercept estimates as well as the log odds estimates using any of the four ordinal regression models. Estimating the intercept accurately may be important when the goal is to predict or to calculate the absolute risk or risk difference. While both SM and adjacent-category model produced unbiased log odds estimates with and without weights, the standard error estimates from the weighted models were more accurate. The efficiency of the estimates varied across all weighted models, especially when many of the ordinal categories had small prevalence.

Despite its robustness to sampling variability and other useful properties, the SM is not commonly used in practice. One possible reason is the lack of familiarity among researchers and practitioners with this model, as it is not as widely known as other models for ordinal outcome variables. Another possible reason is the limited availability of the procedure or function to directly estimate the SM in commonly used statistical software packages. For example, the VGAM package in R^16^ can fit the SM, but without addressing the ordinal constraint. Moreover, the SM has some inferential challenges such as the intrinsic non-linear structure of the predictor and the requirement of multiple constraints in the parameter space to ensure identifiability.

The baseline-category logit model (or multinomial regression model) is another model for discrete outcomes of more than two categories, and under ODS, we can obtain consistent log odds estimates for the population without weights.^13^ We did not include the baseline-category logit model in this article because it does not consider the ordinal nature of the outcome and suffers from a lack of parsimony compared to the SM and the adjacent-category model. However, if the outcome categories are nominal and if the sampling probabilities are unknown, then the baseline-category logit model is useful.

We note that the models in our simulation study only had two independent covariates; including a larger number of covariates where some of them may be correlated with each other warrants additional exploration. We also note that we investigated the case where sampling is dependent only on the outcome. Therefore, results may be different when sampling is also dependent on covariates such as a two-phase design. In our case study in which we applied outcome-dependent sampling to the knee OA study, we repeated the sampling 200 times to empirically quantify the variability introduced by the sampling scheme. However, in a realistic setting, the outcome-dependent procedure will only be performed once with a feasible sample size without any knowledge of the true effect of the expensive variable in the original cohort.

Future work includes identifying sampling proportions that minimize the asymptotic variance of the parameter of interest under ODS and two-phase designs. Indeed, previous work by Espin-Garcia et al.^36^ suggests that optimal sampling proportions can be obtained via constrained non-linear optimization approaches. Alternatively, stochastic optimization techniques, such as genetic algorithms, can be implemented to identify near-optimal designs in different settings. Extending semiparametric inference in two-phase studies to ordinal outcomes with misclassification errors is another research avenue.^37^ This is particularly relevant for OA, where radiographic imaging is subject to error.^38^

Imposing the ordinal constraint on the ϕ parameters of the SM during likelihood maximization deserves further investigation. The rrvglm() function in the R VGAM package we used does not impose such a constraint. This means that it is possible to estimate ${\hat{\phi }}_{k+1}>{\hat{\phi }}_{k}$ if K>3 which leads to a model formulation that is incompatible with the ordinal nature of the data. This unique feature of the SM is useful in determining the order of the categories or combining the categories; however, some might argue that such a model is more useful for nominal variables than ordinal variables. To impose the constraint, Fernández et al.^39^ proposed and implemented a method in the OSM package, which is currently under development.

In conclusion, unbiased and efficient estimates from ordinal models can be obtained with appropriate weights under ODS when the outcome is ordinal. When the sampling depends only on the outcome and the sampling fractions are known, then the inverse of the sampling probability can be used as weights. However, when the sampling probability is unknown, only the SM and AC can yield unbiased log odds estimates.

## Supplemental Material

sj-pdf-1-smm-10.1177_09622802241282091 - Supplemental material for Applying survey weights to ordinal regression models for improved inference in outcome-dependent samples with ordinal outcomes Supplemental material, sj-pdf-1-smm-10.1177_09622802241282091 for Applying survey weights to ordinal regression models for improved inference in outcome-dependent samples with ordinal outcomes by Aya A Mitani, Osvaldo Espin-Garcia, Daniel Fernández and Victoria Landsman in Statistical Methods in Medical Research

## Appendix I

Why the log odds estimates are preserved under ODS with SM and AC.

For SM, continuing equation (9)

$$\begin{array}{rl} \pi_{ik}^{s} & =\frac{f_{k}\pi_{ik}}{\sum_{j=1}^{K}\left\{f_{j}\pi_{ij}\right\}}\\ & =f_{k}\frac{\exp\;\left(\alpha_{k}+X_{i}\beta \phi_{k}\right)}{1+\sum_{h=1}^{K-1}\exp\;\left(\alpha_{h}+X_{i}\beta \phi_{h}\right)}/\sum_{j=1}^{K}f_{j}\frac{\exp\;\left(\alpha_{j}+X_{i}\beta \phi_{j}\right)}{1+\sum_{h=1}^{K-1}\exp\;\left(\alpha_{h}+X_{i}\beta \phi_{h}\right)}\\ & =\frac{f_{k}\exp\;(\alpha_{k}+X_{i}\beta \phi_{k})}{f_{K}+\sum_{j=1}^{K-1}f_{j}\exp\;(\alpha_{j}+X_{i}\beta \phi_{j})}\\ & =\frac{\frac{f_{k}}{f_{K}}\exp\;(\alpha_{k}+X_{i}\beta \phi_{k})}{1+\sum_{j=1}^{K-1}\frac{f_{j}}{f_{K}}\exp\;(\alpha_{j}+X_{i}\beta \phi_{j})}\\ & =\frac{\exp\;(\alpha_{k}+\ln\;\frac{f_{k}}{f_{K}}+X_{i}\beta \phi_{k})}{1+\sum_{j=1}^{K-1}\exp\;(\alpha_{j}+\ln\;\frac{f_{j}}{f_{K}}+X_{i}\beta \phi_{j})}\\ & =\frac{\exp\;(\alpha_{k}^{s}+X_{i}\beta \phi_{k})}{1+\sum_{j=1}^{K-1}\exp\;(\alpha_{j}^{s}+X_{i}\beta \phi_{j})} \end{array}$$

Reassigning the indices to the original h , we obtain equation (10)

$$\pi_{ik}^{s}=\frac{\exp\;(\alpha_{k}^{s}+X_{i}\beta \phi_{k})}{1+\sum_{h=1}^{K-1}\exp\;(\alpha_{h}^{s}+X_{i}\beta \phi_{h})}$$

where $\alpha_{k}^{s}=\alpha_{k}+\ln\;(f_{k}/f_{K})$ and $\alpha_{h}^{s}=\alpha_{h}+\ln\;(f_{h}/f_{K})$ .

Similarly for AC,

$$\begin{array}{rl} \pi_{ik}^{s} & =\frac{f_{k}\pi_{ik}}{\sum_{j=1}^{K}\left\{f_{j}\pi_{ij}\right\}}\\ & =f_{k}\exp\;\left(\sum_{h=k}^{K-1}\alpha_{h}+\beta^{\prime }X_{i}\right)/\left\{\sum_{j=1}^{K-1}f_{j}\exp\;\left(\sum_{j=k}^{K-1}\alpha_{j}+\beta^{\prime }X_{i}\right)+f_{K}\right\}\\ & =\frac{\frac{f_{k}}{f_{K}}\exp\;(\sum_{h=k}^{K-1}\alpha_{h}+\beta^{\prime }X_{i})}{1+\sum_{j=1}^{K-1}\frac{f_{j}}{f_{K}}\exp\;(\sum_{l=j}^{K-1}\alpha_{l}+\beta^{\prime }X_{i})}\\ & =\frac{\exp\;(\sum_{h=k}^{K-1}\alpha_{h}+\ln\;\frac{f_{k}}{f_{K}}+\beta^{\prime }X_{i})}{1+\sum_{j=1}^{K-1}\exp\;(\sum_{l=j}^{K-1}\alpha_{l}+\ln\;\frac{f_{j}}{f_{K}}+\beta^{\prime }X_{i})} \end{array}$$

Reassigning the indices, we obtain equation (11)

$$\pi_{ik}^{s}=\frac{\exp\;(\alpha_{h}^{s}+\beta^{\prime }X_{i})}{1+\sum_{l=1}^{K-1}\exp\;(\alpha_{l}^{s}+\beta^{\prime }X_{i})},$$

where $\alpha_{h}^{s}=\sum_{h=k}^{K-1}\alpha_{h}+\ln\;(f_{k}/f_{K})$ and $\alpha_{l}^{s}=\sum_{l=h}^{K-1}\alpha_{h}+\ln\;(f_{h}/f_{K})$ .

## Appendix II

Special case for CR when $f_{2}=f_{3}=\cdots =f_{K}:=f^{*}$ . For k=1 ,

$$\begin{array}{rl} \pi_{i1}^{s} & =\frac{f_{1}\pi_{i1}}{\sum_{j=1}^{K}\left\{f_{j}\pi_{ij}\right\}}\\ & =\frac{f_{1}\pi_{i1}}{f_{1}\pi_{i1}+f^{*}(1-\pi_{i1})}\\ & =\frac{f_{1}\frac{\exp\;(\alpha_{1}+\beta^{\prime }X_{i})}{1+\exp\;(\alpha_{1}+\beta^{\prime }X_{i})}}{f_{1}\frac{\exp\;(\alpha_{1}+\beta^{\prime }X_{i})}{1+\exp\;(\alpha_{1}+\beta^{\prime }X_{i})}+f^{*}\frac{1}{1+\exp\;(\alpha_{1}+\beta^{\prime }X_{i})}}\\ & =\frac{\frac{f_{1}}{f^{*}}\exp\;(\alpha_{1}+\beta^{\prime }X_{i})}{\frac{f_{1}}{f^{*}}\exp\;(\alpha_{1}+\beta^{\prime }X_{i})+\frac{f^{*}}{f^{*}}}\\ & =\frac{\exp\;(\alpha_{1}+\ln\;\frac{f_{1}}{f^{*}}+\beta^{\prime }X_{i})}{1+\exp\;(\alpha_{1}+\ln\;\frac{f_{1}}{f^{*}}+\beta^{\prime }X_{i})}\\ & =\frac{\exp\;(\alpha_{1}^{*}+\beta^{\prime }X_{i})}{1+\exp\;(\alpha_{1}^{*}+\beta^{\prime }X_{i})}, \end{array}$$

and for k>1 ,

$$\begin{array}{rl} \pi_{ik}^{s} & =\frac{f_{k}\pi_{ik}}{\sum_{j=1}^{K}\left\{f_{j}\pi_{ij}\right\}}\\ & =\frac{f^{*}\pi_{ik}}{f_{1}\pi_{i1}+f^{*}(1-\pi_{i1})}\\ & =\frac{f^{*}\frac{\exp\;(\alpha_{k}+\beta^{\prime }X_{i})}{\prod_{h=1}^{k}\left\{1+\exp\;(\alpha_{h}+\beta^{\prime }X_{i})\right\}}}{f_{1}\frac{\exp\;(\alpha_{1}+\beta^{\prime }X_{i})}{1+\exp\;(\alpha_{1}+\beta^{\prime }X_{i})}+f^{*}\frac{1}{1+\exp\;(\alpha_{1}+\beta^{\prime }X_{i})}}\\ & =\frac{\frac{f^{*}}{f^{*}}\frac{\exp\;(\alpha_{k}+\beta^{\prime }X_{i})}{\prod_{h=2}^{k}\left\{1+\exp\;(\alpha_{h}+\beta^{\prime }X_{i})\right\}}}{\frac{f_{1}}{f^{*}}\exp\;(\alpha_{1}+\beta^{\prime }X_{i})+\frac{f^{*}}{f^{*}}}\\ & =\frac{\exp\;(\alpha_{k}+\beta^{\prime }X_{i})}{\left\{1+\exp\;(\alpha_{1}+\ln\;\frac{f_{1}}{f^{*}}+\beta^{\prime }X_{i})\right\}\prod_{h=2}^{k}\left\{1+\exp\;(\alpha_{h}+\beta^{\prime }X_{i})\right\}}\\ & =\frac{\exp\;(\alpha_{k}+\beta^{\prime }X_{i})}{\left\{1+\exp\;(\alpha_{1}^{*}+\beta^{\prime }X_{i})\right\}\prod_{h=2}^{k}\left\{1+\exp\;(\alpha_{h}+\beta^{\prime }X_{i})\right\}}, \end{array}$$

which is equivalent to equation (6), where $\alpha_{1}^{*}=\alpha_{1}+\ln\;(f_{1}/f^{*})$ .
