# Supplementary material for: Applying survey weights to ordinal regression models for improved inference in outcome-dependent samples with ordinal outcomes
Source: Stat Methods Med Res. 2024 Oct 23;33(11-12):2007–26. doi: 10.1177/09622802241282091 (PMC11577697; doi:10.1177/09622802241282091)
Supplement: sj-pdf-1-smm-10.1177_09622802241282091 - Supplemental material for Applying survey weights to ordinal regression models for improved inference in outcome-dependent samples with ordinal outcomes [file sj-pdf-1-smm-10.1177_09622802241282091.pdf]

Supporting materials for “Applying survey-weighted proportional odds models for improved inference in outcome-dependent samples with ordinal outcomes”

Aya A. Mitani, Osvaldo Espin-Garcia, Daniel Fernández, Victoria Landsman

Table S1: Percent mean relative bias of the unweighted estimators under ODS: RB($\hat{\boldsymbol{\theta}}_u$); and percent mean relative bias of the weighted estimators under ODS: RB($\hat{\boldsymbol{\theta}}_w$) from Scenarios L(i), L(ii), L(iii), L(iv), and L(v) with $K = 5$.

		Scenario L(i)			Scenario L(ii)			Scenario L(iii)			Scenario L(iv)			Scenario L(v)		
Model	$\boldsymbol{\theta}$	True	RB($\hat{\boldsymbol{\theta}}_u$)	RB($\hat{\boldsymbol{\theta}}_w$)	True	RB($\hat{\boldsymbol{\theta}}_u$)	RB($\hat{\boldsymbol{\theta}}_w$)	True	RB($\hat{\boldsymbol{\theta}}_u$)	RB($\hat{\boldsymbol{\theta}}_w$)	True	RB($\hat{\boldsymbol{\theta}}_u$)	RB($\hat{\boldsymbol{\theta}}_w$)	True	RB($\hat{\boldsymbol{\theta}}_u$)	RB($\hat{\boldsymbol{\theta}}_w$)
SM	α_1	0.9	-173.3	-0.2	-0.7	3.3	2.9	-1.6	-53.2	1.6	-2.4	-66.1	1.6	-3.4	-74.8	0.5
SM	α_2	1.2	-128.3	0.9	-0.5	-23.7	0.9	-1.4	-70.9	1.1	-2.2	-78.3	1.1	-3.2	-82.5	0.7
SM	α_3	1.3	-113.0	0.7	-0.4	-38.8	0.6	-1.3	-78.9	1.1	-2.2	-83.8	0.9	-3.2	-86.1	0.4
SM	α_4	1.5	-103.0	0.5	-0.2	-52.5	1.3	-1.2	-85.8	1.0	-2.1	-87.6	1.3	-3.1	-88.5	0.4
SM	β_1	3.5	0.9	0.3	2.9	1.7	1.8	2.6	2.7	2.7	2.4	3.2	3.8	2.4	0.7	1.7
SM	$\phi_2\beta_1$	2.5	0.1	-0.9	2.0	1.1	1.2	1.7	2.9	3.3	1.7	3.4	4.5	1.7	1.5	3.3
SM	$\phi_3\beta_1$	1.9	0.5	-0.9	1.4	0.9	1.0	1.3	4.0	4.8	1.3	3.2	4.9	1.5	0.6	2.4
SM	$\phi_4\beta_1$	1.1	1.4	-1.5	0.9	1.5	1.7	0.9	4.5	6.2	1.0	5.4	8.2	1.3	1.0	3.3
SM	β_2	-1.7	1.6	0.8	-1.4	1.0	1.1	-1.3	1.4	1.4	-1.2	1.5	1.3	-1.2	0.9	0.7
SM	$\phi_2\beta_2$	-1.3	0.6	-0.7	-1.0	0.4	0.4	-0.9	2.0	2.4	-0.8	1.8	2.2	-0.9	1.5	2.2
SM	$\phi_3\beta_2$	-1.0	1.5	-0.2	-0.7	-0.1	-0.0	-0.7	3.0	3.8	-0.7	1.5	2.2	-0.7	0.6	1.5
SM	$\phi_4\beta_2$	-0.6	2.2	-0.8	-0.4	-0.1	0.0	-0.5	3.3	5.0	-0.5	3.5	5.4	-0.6	0.6	2.1
AC	α_1	1.5	-102.9	0.3	-0.2	-63.0	-1.3	-1.2	-91.6	-0.3	-2.0	-93.9	-0.4	-2.9	-94.8	0.1
AC	α_2	-0.2	-35.5	1.1	-0.2	-11.5	0.6	-0.2	-1.7	-0.3	-0.2	11.0	-0.2	-0.2	19.8	2.0
AC	α_3	-0.2	6.5	3.5	-0.2	14.7	2.6	-0.2	17.7	-0.1	-0.2	20.3	0.1	-0.2	33.8	5.9
AC	α_4	-0.2	80.3	6.6	-0.1	99.5	3.6	-0.1	132.3	6.4	-0.1	126.1	4.0	-0.2	98.6	8.8
AC	β_1	0.8	1.5	1.7	0.7	0.8	0.8	0.6	-0.1	0.1	0.6	0.2	1.2	0.6	1.6	2.8
AC	β_2	-0.4	0.1	0.3	-0.3	0.9	0.9	-0.3	0.2	0.1	-0.3	1.8	2.9	-0.3	-0.3	0.7
CR	α_1	2.7	-56.4	0.4	1.0	10.0	-0.1	0.1	1171.7	-2.2	-0.7	-250.9	-1.0	-1.7	-159.4	0.1
CR	α_2	0.8	6.1	0.6	0.8	-2.0	-0.8	0.8	-4.7	-0.8	0.8	-6.3	0.2	0.7	-6.6	-1.7
CR	α_3	0.4	-11.9	0.2	0.4	-18.0	-2.4	0.4	-22.0	-2.9	0.3	-24.3	-1.0	0.3	-39.2	-6.6
CR	α_4	-0.4	33.9	2.1	-0.4	35.8	4.1	-0.4	40.1	5.4	-0.4	33.1	1.9	-0.5	33.4	4.8
CR	β_1	1.3	-1.8	0.1	1.3	-1.4	1.2	1.2	-1.6	1.5	1.2	-3.2	0.3	1.3	-2.5	2.0
CR	β_2	-0.7	-1.1	1.0	-0.6	-2.0	0.5	-0.6	-1.6	0.9	-0.6	-2.7	1.0	-0.7	-2.7	1.3
CM	α_1	-2.7	-57.0	0.2	-1.0	11.0	0.1	0.0	-56435.3	-696.5	0.8	-223.4	-0.8	1.8	-153.1	0.6
CM	α_2	-0.5	-85.3	0.0	0.0	-736.2	82.1	0.6	-101.1	-1.5	1.3	-98.8	-0.2	2.2	-97.6	0.7
CM	α_3	0.5	66.0	1.0	0.8	3.4	0.9	1.3	-32.4	-0.5	1.8	-51.9	0.2	2.6	-65.9	0.6
CM	α_4	1.6	20.8	0.7	1.8	5.5	0.6	2.1	-11.7	-0.1	2.6	-27.2	0.2	3.3	-42.8	0.6
CM	β_1	-1.7	7.9	1.1	-1.7	-1.8	0.7	-1.7	-13.1	-0.3	-1.7	-23.1	1.1	-1.8	-33.6	2.0
CM	β_2	0.8	8.1	1.1	0.8	-2.0	0.5	0.8	-12.0	1.3	0.8	-22.0	2.3	0.9	-33.9	0.7

ODS: outcome-dependent sampling; SM: stereotype model; AC: adjacent-category logit model;
CR: continuation-ratio logit model; CM: cumulative logit model.

Table S2: Percent mean relative bias of the unweighted estimators under ODS: RB($\hat{\boldsymbol{\theta}}_u$); and percent mean relative bias of the weighted estimators under ODS: RB($\hat{\boldsymbol{\theta}}_w$) from Scenarios M(i), M(ii), M(iii), M(iv), and M(v) with $K = 5$.

		Scenario M(i)			Scenario M(ii)			Scenario M(iii)			Scenario M(iv)			Scenario M(v)		
Model	$\boldsymbol{\theta}$	True	RB($\hat{\boldsymbol{\theta}}_u$)	RB($\hat{\boldsymbol{\theta}}_w$)	True	RB($\hat{\boldsymbol{\theta}}_u$)	RB($\hat{\boldsymbol{\theta}}_w$)	True	RB($\hat{\boldsymbol{\theta}}_u$)	RB($\hat{\boldsymbol{\theta}}_w$)	True	RB($\hat{\boldsymbol{\theta}}_u$)	RB($\hat{\boldsymbol{\theta}}_w$)	True	RB($\hat{\boldsymbol{\theta}}_u$)	RB($\hat{\boldsymbol{\theta}}_w$)
SM	α_1	-0.7	0.2	0.2	-0.7	0.7	0.5	-0.8	-0.5	0.3	-0.9	2.1	2.5	-1.0	1.2	3.7
SM	α_2	-0.4	-29.7	-2.4	-0.5	-24.6	0.0	-0.6	-23.5	-0.2	-0.7	-17.8	2.9	-0.9	-17.3	1.9
SM	α_3	-2.1	-90.2	-0.3	-0.4	-40.6	-1.0	0.6	-138.4	0.6	1.4	-117.9	-0.6	2.3	-110.5	-0.5
SM	α_4	-0.3	-49.5	-2.2	-0.2	-55.8	-1.2	-0.2	-65.7	-1.1	-0.2	-73.5	1.5	-0.2	-92.1	0.8
SM	β_1	2.6	1.3	1.4	2.9	1.5	1.6	3.2	1.3	1.5	3.5	2.0	2.6	4.1	2.2	3.4
SM	$\phi_2\beta_1$	1.7	0.6	0.7	2.0	2.3	2.3	2.3	1.4	1.6	2.8	2.7	3.2	3.5	1.6	2.7
SM	$\phi_3\beta_1$	1.3	0.9	1.1	1.4	1.5	1.5	1.6	1.4	1.6	1.8	3.1	3.8	2.0	3.4	4.6
SM	$\phi_4\beta_1$	1.0	1.7	2.0	0.9	2.1	2.3	0.8	1.5	1.8	0.7	4.5	5.6	0.6	0.9	2.3
SM	β_2	-1.3	2.7	2.7	-1.4	2.5	2.5	-1.6	2.3	2.5	-1.8	1.3	1.7	-2.0	2.3	2.6
SM	$\phi_2\beta_2$	-0.8	2.0	1.9	-1.0	3.3	3.2	-1.2	2.4	2.5	-1.4	2.1	2.4	-1.7	1.6	1.8
SM	$\phi_3\beta_2$	-0.7	2.0	2.1	-0.7	2.0	2.0	-0.8	2.1	2.2	-0.9	2.4	2.9	-1.0	3.4	3.8
SM	$\phi_4\beta_2$	-0.5	3.2	3.2	-0.4	3.4	3.5	-0.4	1.9	2.3	-0.4	3.8	4.6	-0.3	0.7	1.6
AC	α_1	-0.2	-62.3	-1.4	-0.2	-63.8	-1.5	-0.2	-64.8	-0.0	-0.2	-67.4	-0.0	-0.2	-85.9	-5.5
AC	α_2	-1.9	-93.5	0.0	-0.2	-12.6	-1.5	0.8	-119.9	-0.7	1.6	-111.5	-0.6	2.5	-107.5	0.1
AC	α_3	1.6	-110.9	-0.0	-0.2	13.7	1.0	-1.2	-79.3	0.7	-2.0	-85.3	0.8	-3.0	-88.3	0.1
AC	α_4	-0.1	123.9	5.3	-0.1	98.0	2.1	-0.2	77.2	7.1	-0.2	64.8	6.6	-0.4	41.0	1.4
AC	β_1	0.6	1.0	1.1	0.7	0.4	0.4	0.8	1.5	1.9	0.9	2.2	2.6	1.1	-0.0	1.5
AC	β_2	-0.3	1.0	1.2	-0.3	1.1	1.1	-0.4	0.3	0.6	-0.5	0.3	0.7	-0.5	1.9	2.8
CR	α_1	0.8	44.9	-0.3	1.0	9.7	-0.4	1.4	-18.0	-0.1	1.8	-38.6	0.1	2.5	-56.1	-0.2
CR	α_2	0.5	76.2	-1.8	0.8	-2.4	-1.0	1.2	-39.9	-0.2	1.8	-59.7	0.2	2.5	-74.2	-0.3
CR	α_3	2.2	-82.7	-0.4	0.4	-18.5	-3.2	-0.6	-138.3	1.0	-1.5	-110.6	0.3	-2.5	-100.7	0.7
CR	α_4	-0.3	20.3	5.5	-0.4	37.4	5.7	-0.4	45.7	2.6	-0.5	47.2	2.3	-0.6	41.7	3.6
CR	β_1	1.3	-11.8	2.0	1.3	-0.6	1.9	1.3	6.6	0.5	1.4	11.7	0.4	1.7	15.7	2.3
CR	β_2	-0.6	-12.1	1.4	-0.6	-1.6	1.0	-0.7	6.2	0.0	-0.7	11.6	0.2	-0.8	16.0	1.6
CM	α_1	-0.8	46.8	-0.2	-1.0	11.2	0.2	-1.3	-17.9	-0.0	-1.8	-39.1	-0.3	-2.5	-54.9	-0.2
CM	α_2	0.3	-118.8	2.2	0.0	-798.1	24.9	-0.4	-101.6	-0.7	-1.0	-104.3	-1.0	-1.7	-103.3	-0.4
CM	α_3	0.5	63.0	1.7	0.8	3.0	0.4	1.3	-28.5	0.8	1.8	-46.1	0.9	2.7	-59.9	0.9
CM	α_4	1.6	17.3	1.0	1.8	5.4	0.3	2.2	-7.6	0.6	2.6	-20.0	0.7	3.4	-34.1	0.8
CM	β_1	-1.6	-10.0	1.3	-1.7	-2.1	0.5	-1.7	8.5	1.2	-1.8	16.8	1.7	-1.9	22.6	2.5
CM	β_2	0.8	-10.9	0.3	0.8	-2.5	0.0	0.8	8.4	1.0	0.9	15.9	0.2	1.0	25.1	2.6

ODS: outcome-dependent sampling; SM: stereotype model; AC: adjacent-category logit model;
CR: continuation-ratio logit model; CM: cumulative logit model.

Table S3: Percent mean relative bias of the unweighted estimators under ODS: RB($\hat{\boldsymbol{\theta}}_u$); and percent mean relative bias of the weighted estimators under ODS: RB($\hat{\boldsymbol{\theta}}_w$) from Scenarios H(i), H(ii), H(iii), H(iv), and H(v) with $K = 5$.

		Scenario H(i)			Scenario H(ii)			Scenario H(iii)			Scenario H(iv)			Scenario H(v)		
Model	$\boldsymbol{\theta}$	True	RB($\hat{\boldsymbol{\theta}}_u$)	RB($\hat{\boldsymbol{\theta}}_w$)	True	RB($\hat{\boldsymbol{\theta}}_u$)	RB($\hat{\boldsymbol{\theta}}_w$)	True	RB($\hat{\boldsymbol{\theta}}_u$)	RB($\hat{\boldsymbol{\theta}}_w$)	True	RB($\hat{\boldsymbol{\theta}}_u$)	RB($\hat{\boldsymbol{\theta}}_w$)	True	RB($\hat{\boldsymbol{\theta}}_u$)	RB($\hat{\boldsymbol{\theta}}_w$)
SM	α_1	-2.7	-59.5	0.6	-0.7	3.0	2.5	0.3	-267.9	-9.2	1.2	-136.3	-2.1	2.2	-114.1	0.6
SM	α_2	-0.6	-8.2	1.7	-0.5	-22.3	2.4	-0.4	-38.8	2.1	-0.3	-58.6	0.8	-0.3	-83.1	-4.0
SM	α_3	-0.4	-30.5	0.7	-0.4	-38.3	1.7	-0.3	-48.7	1.9	-0.3	-66.1	-0.2	-0.2	-89.5	-4.6
SM	α_4	-0.3	-45.4	2.5	-0.2	-54.5	-0.4	-0.2	-60.5	1.1	-0.2	-77.7	0.8	-0.2	-95.1	-5.4
SM	β_1	3.5	1.7	1.2	2.9	1.5	1.5	2.6	2.4	2.5	2.4	2.5	3.6	2.4	0.3	0.9
SM	$\phi_2\beta_1$	2.4	1.0	1.7	2.0	2.1	2.1	1.6	2.2	1.3	1.4	2.7	2.3	1.1	1.1	0.0
SM	$\phi_3\beta_1$	1.6	0.9	1.5	1.4	1.4	1.4	1.3	2.0	1.1	1.1	1.9	1.5	0.9	1.1	0.2
SM	$\phi_4\beta_1$	1.0	3.3	4.1	0.9	-0.3	-0.1	0.8	2.2	1.4	0.7	2.3	2.3	0.6	2.3	1.5
SM	β_2	-1.7	2.4	1.3	-1.4	0.8	0.8	-1.3	0.4	0.7	-1.2	2.9	2.9	-1.2	2.9	3.3
SM	$\phi_2\beta_2$	-1.2	1.6	1.7	-1.0	1.4	1.4	-0.8	0.5	-0.2	-0.7	3.4	1.8	-0.6	3.7	2.4
SM	$\phi_3\beta_2$	-0.8	1.4	1.5	-0.7	0.6	0.6	-0.6	-0.2	-0.8	-0.6	2.5	0.9	-0.5	4.3	3.0
SM	$\phi_4\beta_2$	-0.5	3.9	4.2	-0.4	-0.9	-0.7	-0.4	0.8	0.0	-0.4	3.2	1.7	-0.3	4.6	3.7
AC	α_1	-0.2	-55.2	-3.2	-0.2	-62.5	-1.1	-0.2	-71.9	-2.0	-0.2	-82.7	0.4	-0.1	-102.4	-3.2
AC	α_2	-0.2	-4.4	-1.5	-0.2	-10.6	0.9	-0.1	-26.1	0.4	-0.1	-29.0	4.0	-0.1	-58.3	-1.6
AC	α_3	-0.2	36.4	2.9	-0.2	15.8	2.1	-0.2	-3.0	1.5	-0.1	-8.9	6.6	-0.1	-20.2	4.6
AC	α_4	-1.9	-81.1	0.3	-0.1	101.4	6.7	0.9	-124.6	-1.0	1.7	-111.0	-0.8	2.6	-106.1	-0.3
AC	β_1	0.8	0.6	0.7	0.7	1.3	1.3	0.6	0.8	1.0	0.6	1.4	2.8	0.6	0.6	2.9
AC	β_2	-0.4	1.9	2.1	-0.3	0.8	0.8	-0.3	1.1	0.9	-0.3	0.9	1.0	-0.3	2.3	3.6
CR	α_1	0.8	27.0	0.2	1.0	11.1	1.0	1.4	-10.3	-0.0	1.8	-28.7	-0.2	2.5	-46.5	-0.4
CR	α_2	0.5	35.1	-0.8	0.8	-0.6	0.8	1.3	-27.9	-0.4	1.8	-45.7	-0.5	2.5	-60.0	-0.5
CR	α_3	-0.2	-152.0	4.5	0.4	-14.7	0.7	1.0	-55.7	-0.7	1.6	-68.6	-0.7	2.5	-77.0	-0.7
CR	α_4	-2.2	-64.4	0.7	-0.4	33.0	0.6	0.7	-147.9	-2.1	1.5	-116.1	-1.3	2.5	-107.1	-0.8
CR	β_1	1.3	22.9	1.0	1.3	-2.4	0.1	1.3	-20.9	1.0	1.4	-33.6	2.0	1.6	-44.1	2.3
CR	β_2	-0.7	23.1	1.4	-0.6	-0.5	2.1	-0.6	-21.1	0.7	-0.7	-33.6	2.0	-0.8	-43.8	1.4
CM	α_1	-0.7	33.8	-0.6	-1.0	11.2	0.3	-1.3	-11.0	0.1	-1.8	-28.6	0.2	-2.5	-46.0	-0.1
CM	α_2	0.3	-75.3	3.2	0.0	-779.8	18.8	-0.4	-68.6	-0.8	-1.0	-76.1	-0.1	-1.8	-81.7	-0.4
CM	α_3	1.4	-27.8	1.3	0.8	3.1	0.6	0.2	249.1	2.9	-0.4	-243.1	-1.2	-1.3	-139.8	-0.8
CM	α_4	3.5	-41.3	0.7	1.8	5.4	0.4	0.8	114.5	1.1	0.0	14414.5	82.3	-1.0	-260.1	-1.2
CM	β_1	-1.7	8.9	1.8	-1.7	-2.2	0.3	-1.7	-12.2	0.3	-1.7	-22.8	1.4	-1.8	-33.7	1.9
CM	β_2	0.8	7.3	0.6	0.8	-2.2	0.4	0.8	-13.1	0.0	0.8	-22.2	2.4	0.9	-32.6	2.2

ODS: outcome-dependent sampling; SM: stereotype model; AC: adjacent-category logit model;

CR: continuation-ratio logit model; CM: cumulative logit model.

Table S4: Percent mean relative bias of the unweighted estimators under ODS: RB($\hat{\boldsymbol{\theta}}_u$); and percent mean relative bias of the weighted estimators under ODS: RB($\hat{\boldsymbol{\theta}}_w$) from Scenarios L(i), L(ii), L(iii), L(iv), and L(v) with $K = 3$.

		Scenario L(i)			Scenario L(ii)			Scenario L(iii)			Scenario L(iv)			Scenario L(v)		
Model	$\boldsymbol{\theta}$	True	RB($\hat{\boldsymbol{\theta}}_u$)	RB($\hat{\boldsymbol{\theta}}_w$)	True	RB($\hat{\boldsymbol{\theta}}_u$)	RB($\hat{\boldsymbol{\theta}}_w$)	True	RB($\hat{\boldsymbol{\theta}}_u$)	RB($\hat{\boldsymbol{\theta}}_w$)	True	RB($\hat{\boldsymbol{\theta}}_u$)	RB($\hat{\boldsymbol{\theta}}_w$)	True	RB($\hat{\boldsymbol{\theta}}_u$)	RB($\hat{\boldsymbol{\theta}}_w$)
SM	α_1	0.8	-161.3	-1.7	-0.4	37.8	2.1	-1.2	-51.2	0.9	-2.1	-67.5	0.9	-3.5	-76.2	0.8
SM	α_2	1.2	-110.4	0.4	-0.1	45.1	3.1	-1.0	-75.5	0.3	-1.9	-81.6	0.9	-3.3	-83.8	0.9
SM	β_1	2.7	1.2	1.0	2.2	1.7	1.6	2.1	1.9	1.8	2.1	1.5	2.0	2.2	1.4	2.4
SM	$\phi_2\beta_1$	1.3	0.3	-2.0	1.1	3.0	2.8	1.1	1.3	1.9	1.3	2.5	3.7	1.6	2.5	3.9
SM	β_2	-1.3	1.5	0.3	-1.1	1.5	1.5	-1.1	1.7	1.9	-1.0	0.4	0.7	-1.1	0.7	1.1
SM	$\phi_2\beta_2$	-0.6	0.6	-2.6	-0.6	2.7	2.5	-0.6	1.0	1.9	-0.6	1.4	2.5	-0.8	1.8	2.6
AC	α_1	1.2	-110.9	0.1	-0.1	24.6	-8.9	-1.0	-77.1	-0.6	-1.8	-85.4	-0.4	-3.2	-88.6	0.3
AC	α_2	-0.3	21.1	4.5	-0.3	33.8	0.2	-0.3	44.7	1.9	-0.3	47.9	2.7	-0.4	43.6	6.4
AC	β_1	1.3	1.9	1.7	1.1	0.7	0.6	1.1	0.5	0.6	1.1	0.8	1.0	1.2	2.0	3.0
AC	β_2	-0.7	0.6	0.6	-0.6	3.1	3.0	-0.5	1.5	1.5	-0.5	1.8	2.3	-0.6	1.3	1.5
CR	α_1	1.8	-75.0	0.1	0.5	-18.9	-0.6	-0.4	-195.5	-1.6	-1.2	-123.5	0.2	-2.6	-105.8	0.1
CR	α_2	-0.4	14.6	1.9	-0.4	24.2	4.7	-0.4	23.7	1.2	-0.5	24.0	3.2	-0.6	18.1	3.6
CR	β_1	1.5	0.4	0.8	1.4	0.8	1.9	1.4	-1.2	-0.0	1.5	-0.5	1.3	1.6	-0.3	1.7
CR	β_2	-0.8	-0.8	-0.0	-0.7	-0.3	0.8	-0.7	-0.6	0.7	-0.7	-0.7	0.6	-0.8	0.2	2.0
CM	α_1	-1.8	-75.4	1.0	-0.5	-18.5	1.9	0.4	-176.9	-0.5	1.3	-119.2	-0.1	2.7	-104.7	0.8
CM	α_2	0.6	75.7	-0.8	1.0	10.8	0.3	1.5	-21.4	0.3	2.2	-42.5	0.2	3.4	-59.8	0.7
CM	β_1	-1.7	7.1	0.1	-1.6	1.0	1.2	-1.6	-6.2	0.5	-1.7	-13.3	0.7	-1.9	-19.9	2.5
CM	β_2	0.8	8.8	2.2	0.8	2.6	2.9	0.8	-6.8	-0.1	0.8	-12.5	1.3	1.0	-18.6	1.5

ODS: outcome-dependent sampling; SM: stereotype model; AC: adjacent-category logit model;

CR: continuation-ratio logit model; CM: cumulative logit model.

Table S5: Percent mean relative bias of the unweighted estimators under ODS: RB($\hat{\boldsymbol{\theta}}_u$); and percent mean relative bias of the weighted estimators under ODS: RB($\hat{\boldsymbol{\theta}}_w$) from Scenarios M(i), M(ii), M(iii), M(iv), and M(v) with $K = 3$.

		Scenario M(i)			Scenario M(ii)			Scenario M(iii)			Scenario M(iv)			Scenario M(v)		
Model	$\boldsymbol{\theta}$	True	RB($\hat{\boldsymbol{\theta}}_u$)	RB($\hat{\boldsymbol{\theta}}_w$)	True	RB($\hat{\boldsymbol{\theta}}_u$)	RB($\hat{\boldsymbol{\theta}}_w$)	True	RB($\hat{\boldsymbol{\theta}}_u$)	RB($\hat{\boldsymbol{\theta}}_w$)	True	RB($\hat{\boldsymbol{\theta}}_u$)	RB($\hat{\boldsymbol{\theta}}_w$)	True	RB($\hat{\boldsymbol{\theta}}_u$)	RB($\hat{\boldsymbol{\theta}}_w$)
SM	α_1	-0.4	-0.1	0.3	-0.5	0.5	1.4	-0.6	0.6	0.5	-0.8	1.4	1.1	-1.0	1.7	4.6
SM	α_2	-1.7	-90.8	-0.1	-0.4	-57.3	-0.6	0.4	-155.7	0.4	1.2	-118.5	0.4	2.4	-109.6	-0.5
SM	β_1	1.8	0.3	0.7	2.1	1.7	1.8	2.6	1.0	1.1	3.1	1.8	2.3	4.0	2.0	3.0
SM	$\phi_2\beta_1$	0.9	-0.6	-0.2	1.1	1.4	1.4	1.3	1.7	1.8	1.5	1.8	2.2	2.0	1.9	2.8
SM	β_2	-0.9	1.2	1.3	-1.1	2.2	2.2	-1.3	2.0	2.2	-1.6	2.8	3.3	-2.0	1.2	1.4
SM	$\phi_2\beta_2$	-0.4	0.5	0.4	-0.5	1.9	1.9	-0.6	2.5	2.6	-0.8	2.8	3.3	-1.0	1.1	1.3
AC	α_1	-1.7	-90.7	-0.0	-0.4	-57.2	-1.0	0.4	-155.5	2.8	1.2	-120.0	-0.5	2.4	-109.1	-0.1
AC	α_2	1.3	-121.5	-0.8	-0.1	243.9	1.9	-1.0	-56.7	0.5	-1.9	-70.7	1.1	-3.4	-76.9	0.8
AC	β_1	0.9	1.1	1.3	1.1	0.1	0.1	1.3	1.0	1.0	1.5	1.6	2.1	2.0	1.5	2.5
AC	β_2	-0.4	0.7	0.6	-0.5	0.5	0.6	-0.6	2.7	2.8	-0.8	0.8	1.2	-1.0	1.1	2.0
CR	α_1	-0.2	-397.1	1.8	0.3	54.2	1.4	0.7	-52.9	-0.0	1.3	-78.4	-0.2	2.5	-90.1	0.1
CR	α_2	1.2	-127.8	-1.0	-0.2	106.4	3.0	-1.1	-48.5	1.5	-2.0	-64.5	0.7	-3.4	-71.9	0.8
CR	β_1	1.4	-18.3	1.1	1.4	-3.0	0.3	1.5	10.2	2.4	1.7	16.2	1.3	2.0	21.6	3.1
CR	β_2	-0.7	-18.3	1.0	-0.7	-2.6	0.6	-0.7	10.4	2.7	-0.8	15.3	0.3	-1.0	22.3	2.9
CM	α_1	0.2	-305.9	2.9	-0.2	59.1	-3.0	-0.7	-53.5	1.2	-1.3	-79.2	-0.2	-2.5	-91.7	-0.3
CM	α_2	0.6	78.0	1.5	1.0	11.6	1.6	1.5	-19.6	0.2	2.2	-38.2	0.9	3.5	-54.6	1.1
CM	β_1	-1.6	-16.8	0.8	-1.6	-1.7	1.9	-1.7	9.9	0.7	-1.8	23.0	2.2	-2.1	33.5	3.5
CM	β_2	0.8	-17.5	0.1	0.8	-2.9	0.8	0.9	10.8	1.6	0.9	23.0	1.8	1.0	33.2	2.6

ODS: outcome-dependent sampling; SM: stereotype model; AC: adjacent-category logit model;
CR: continuation-ratio logit model; CM: cumulative logit model.

Table S6: Percent mean relative bias of the unweighted estimators under ODS: RB($\hat{\boldsymbol{\theta}}_u$); and percent mean relative bias of the weighted estimators under ODS: RB($\hat{\boldsymbol{\theta}}_w$) from Scenarios H(i), H(ii), H(iii), H(iv), and H(v) with $K = 3$.

		Scenario H(i)			Scenario H(ii)			Scenario H(iii)			Scenario H(iv)			Scenario H(v)		
Model	$\boldsymbol{\theta}$	True	RB($\hat{\boldsymbol{\theta}}_u$)	RB($\hat{\boldsymbol{\theta}}_w$)	True	RB($\hat{\boldsymbol{\theta}}_u$)	RB($\hat{\boldsymbol{\theta}}_w$)	True	RB($\hat{\boldsymbol{\theta}}_u$)	RB($\hat{\boldsymbol{\theta}}_w$)	True	RB($\hat{\boldsymbol{\theta}}_u$)	RB($\hat{\boldsymbol{\theta}}_w$)	True	RB($\hat{\boldsymbol{\theta}}_u$)	RB($\hat{\boldsymbol{\theta}}_w$)
SM	α_1	-2.2	-61.7	0.4	-0.7	-15.8	4.0	0.2	-358.2	-3.5	1.0	-136.4	-1.1	2.4	-112.4	-0.8
SM	α_2	-0.4	-12.2	2.8	-0.3	-26.8	3.8	-0.3	-46.5	-1.3	-0.2	-61.3	0.7	-0.2	-90.8	3.0
SM	β_1	2.7	2.0	1.2	2.3	2.9	2.9	2.1	1.5	1.5	2.1	0.6	1.2	2.2	2.0	3.1
SM	$\phi_2\beta_1$	1.4	2.3	3.4	1.1	3.8	4.0	1.0	2.1	1.3	0.8	2.4	1.7	0.6	1.6	2.1
SM	β_2	-1.3	1.4	0.7	-1.1	1.0	0.9	-1.0	1.6	1.6	-1.0	0.1	0.2	-1.1	1.7	1.8
SM	$\phi_2\beta_2$	-0.7	1.6	2.8	-0.6	1.9	2.1	-0.5	2.6	1.9	-0.4	1.6	0.3	-0.3	0.1	-0.6
AC	α_1	-0.3	-16.0	-0.3	-0.3	-29.3	2.1	-0.3	-43.6	-0.4	-0.2	-57.8	-1.8	-0.2	-78.7	0.0
AC	α_2	-1.9	-70.4	0.9	-0.4	-7.3	4.7	0.4	-170.8	-2.8	1.3	-120.7	-0.8	2.6	-109.5	-0.6
AC	β_1	1.3	1.4	1.6	1.1	2.6	2.6	1.1	1.8	1.9	1.1	1.3	1.8	1.2	2.1	3.0
AC	β_2	-0.7	2.0	1.8	-0.6	0.9	1.0	-0.5	1.4	1.5	-0.5	2.1	2.0	-0.6	1.4	2.1
CR	α_1	-0.2	-221.0	1.4	0.3	44.4	2.6	0.7	-38.7	-1.9	1.3	-61.4	-0.9	2.5	-75.6	-0.5
CR	α_2	-1.9	-62.6	1.3	-0.5	-10.3	0.9	0.4	-204.9	-6.1	1.2	-123.9	-1.6	2.5	-108.4	-0.8
CR	β_1	1.5	16.8	1.9	1.4	1.9	1.0	1.4	-8.6	1.8	1.5	-16.7	2.9	1.9	-24.9	3.1
CR	β_2	-0.8	17.2	1.7	-0.7	3.3	2.4	-0.7	-10.3	-0.5	-0.8	-16.9	2.3	-0.9	-24.2	2.3
CM	α_1	0.2	-201.6	-1.3	-0.2	53.6	0.2	-0.7	-39.2	-0.1	-1.3	-61.4	-0.4	-2.5	-74.6	-0.2
CM	α_2	2.6	-48.9	0.5	1.3	-6.8	0.6	0.4	161.9	1.8	-0.4	-326.4	-2.7	-1.7	-150.3	-0.5
CM	β_1	-1.7	8.4	1.3	-1.6	-0.1	0.3	-1.6	-6.0	0.8	-1.7	-12.8	1.1	-1.9	-19.3	2.2
CM	β_2	0.8	9.2	2.1	0.8	-0.1	0.2	0.8	-6.4	0.9	0.8	-12.7	0.5	1.0	-17.6	2.2

ODS: outcome-dependent sampling; SM: stereotype model; AC: adjacent-category logit model;
CR: continuation-ratio logit model; CM: cumulative logit model.

Table S7: Percent (%) relative error of model-based standard errors and Percent (%) relative error of design-based standard errors of weighted models under ODS from Scenarios L(i), L(ii), L(iii), L(iv), and L(v) with $K = 5$.

		Scenario L(i)		Scenario L(ii)		Scenario L(iii)		Scenario L(iv)		Scenario L(v)	
Model	$\boldsymbol{\theta}$	RE($\hat{\boldsymbol{\theta}}_u$)	RE($\hat{\boldsymbol{\theta}}_w$)	RE($\hat{\boldsymbol{\theta}}_u$)	RE($\hat{\boldsymbol{\theta}}_w$)	RE($\hat{\boldsymbol{\theta}}_u$)	RE($\hat{\boldsymbol{\theta}}_w$)	RE($\hat{\boldsymbol{\theta}}_u$)	RE($\hat{\boldsymbol{\theta}}_w$)	RE($\hat{\boldsymbol{\theta}}_u$)	RE($\hat{\boldsymbol{\theta}}_w$)
SM	α_1	8.1	0.9	6.4	0.9	5.4	-0.3	5.1	-0.1	2.0	-4.7
SM	α_2	14.2	-2.2	18.9	2.2	17.1	0.6	16.1	1.6	5.9	-6.3
SM	α_3	32.7	1.2	31.6	-0.7	25.4	-0.8	23.0	0.6	11.9	-4.2
SM	α_4	69.8	-4.5	56.1	-5.1	38.7	-3.7	29.6	-1.7	16.2	-6.1
SM	β_1	2.1	3.0	-4.3	-1.3	-3.4	-0.6	-0.8	1.2	-8.4	-6.3
SM	$\phi_2\beta_1$	-0.7	1.1	-4.0	-1.1	-3.3	-1.0	-1.2	1.5	-6.5	-5.0
SM	$\phi_3\beta_1$	0.3	2.3	-6.6	-3.9	-3.7	-0.9	0.5	3.5	-7.0	-3.9
SM	$\phi_4\beta_1$	-0.2	2.6	-2.6	0.3	-7.3	-4.4	-2.8	0.2	-6.4	-3.3
SM	β_2	-3.8	0.2	-2.5	0.9	-3.8	-0.1	-1.4	1.5	-2.1	1.6
SM	$\phi_2\beta_2$	-1.9	2.3	-3.1	-0.1	-7.0	-3.9	-5.2	-2.3	0.2	2.9
SM	$\phi_3\beta_2$	-5.5	-0.7	-4.8	-1.9	-6.7	-3.8	-2.9	1.0	-1.8	2.2
SM	$\phi_4\beta_2$	-1.8	1.3	0.5	3.2	-6.9	-3.8	-3.5	0.2	-0.8	2.1
AC	α_1	129.2	-12.0	154.0	-5.4	138.8	-10.1	151.7	-7.1	134.6	-15.1
AC	α_2	126.5	-8.4	144.6	-5.9	132.0	-11.6	142.0	-11.4	121.4	-21.7
AC	α_3	111.6	-6.2	124.5	-7.2	118.6	-7.8	125.3	-10.6	104.7	-18.0
AC	α_4	92.0	-4.9	102.8	-5.8	103.6	-9.1	108.0	-6.0	89.7	-15.5
AC	β_1	-3.2	-2.3	-2.1	-2.1	0.4	-0.5	3.0	0.1	-1.3	-5.6
AC	β_2	-3.0	-2.3	1.4	1.6	-1.5	-1.3	-1.9	-1.9	-0.6	-1.3
CR	α_1	35.5	-5.7	35.5	-2.5	40.6	1.0	34.7	-0.7	33.6	-1.4
CR	α_2	35.4	-3.8	34.3	-2.7	38.3	0.3	33.3	-1.1	30.8	-4.5
CR	α_3	34.6	-4.8	33.0	-3.0	35.8	0.5	31.5	-0.2	27.8	-3.5
CR	α_4	35.7	-5.0	34.2	-2.8	35.9	-0.6	33.9	-0.2	28.3	-5.1
CR	β_1	0.1	-3.7	-0.4	-1.4	1.3	0.5	1.4	1.9	0.1	-1.6
CR	β_2	-1.4	-1.3	-0.7	-1.3	2.3	0.2	-2.0	-3.0	1.2	1.2
CM	α_1	24.0	-3.7	26.5	1.2	20.2	-2.2	15.3	-3.6	15.4	-0.8
CM	α_2	15.6	-1.7	16.5	0.5	11.7	-2.4	7.5	-3.4	7.0	-0.6
CM	α_3	13.7	-1.7	13.5	-0.2	10.0	-2.0	6.3	-3.2	5.8	-0.9
CM	α_4	17.1	-1.8	16.5	-0.3	13.7	-2.2	10.3	-3.3	9.8	-1.4
CM	β_1	-0.3	-1.2	-1.3	-1.6	-0.9	1.0	-4.0	-2.2	-3.2	0.0
CM	β_2	0.5	-1.1	-0.5	-0.7	1.2	-0.4	-6.2	-2.8	0.4	0.5

ODS: outcome-dependent sampling; SM: stereotype model; AC: adjacent-category logit model;

CR: continuation-ratio logit model; CM: cumulative logit model.

Table S8: Percent (%) relative error of model-based standard errors and Percent (%) relative error of design-based standard errors of weighted models under ODS from Scenarios M(i), M(ii), M(iii), M(iv), and M(v) with $K = 5$.

		Scenario M(i)		Scenario M(ii)		Scenario M(iii)		Scenario M(iv)		Scenario M(v)	
Model	$\boldsymbol{\theta}$	RE($\hat{\boldsymbol{\theta}}_u$)	RE($\hat{\boldsymbol{\theta}}_w$)	RE($\hat{\boldsymbol{\theta}}_u$)	RE($\hat{\boldsymbol{\theta}}_w$)	RE($\hat{\boldsymbol{\theta}}_u$)	RE($\hat{\boldsymbol{\theta}}_w$)	RE($\hat{\boldsymbol{\theta}}_u$)	RE($\hat{\boldsymbol{\theta}}_w$)	RE($\hat{\boldsymbol{\theta}}_u$)	RE($\hat{\boldsymbol{\theta}}_w$)
SM	α_1	2.4	-3.1	7.7	1.5	8.4	2.0	4.6	-2.2	14.3	5.6
SM	α_2	18.4	-0.4	20.5	2.6	15.0	-0.3	13.7	-1.1	13.0	3.3
SM	α_3	26.2	-6.0	32.0	-1.0	30.8	-1.5	29.5	-5.1	41.0	2.2
SM	α_4	47.3	-2.0	71.5	2.8	89.1	-1.0	112.0	-10.6	195.4	-16.6
SM	β_1	-7.3	-4.6	-2.3	0.6	-2.7	-0.1	-4.3	-2.1	1.1	3.3
SM	$\phi_2\beta_1$	-4.4	-1.6	-3.0	-0.4	-3.6	-1.4	-1.3	0.1	-3.1	1.2
SM	$\phi_3\beta_1$	-4.8	-2.1	-5.3	-3.0	-5.7	-3.9	-3.8	-3.2	0.0	-0.0
SM	$\phi_4\beta_1$	-1.6	1.0	-0.9	1.8	-3.2	-1.3	0.1	1.3	-3.8	-1.9
SM	β_2	-1.6	1.3	2.5	5.8	-0.2	3.3	-2.6	-1.0	-3.5	-1.4
SM	$\phi_2\beta_2$	-2.5	0.5	-0.0	3.3	-1.8	1.2	-3.2	-1.2	-1.8	-0.3
SM	$\phi_3\beta_2$	-0.2	2.8	-0.3	2.8	-3.0	-1.1	-3.3	-2.7	-1.2	-2.2
SM	$\phi_4\beta_2$	-0.7	2.1	-2.0	0.9	-0.6	1.6	2.1	3.2	-2.6	-1.5
AC	α_1	150.5	-6.8	149.8	-6.4	136.4	-11.3	133.4	-13.3	112.1	-18.2
AC	α_2	144.6	-17.3	146.0	-7.3	129.0	-10.1	131.2	-5.6	114.8	-8.0
AC	α_3	131.2	-16.6	130.0	-3.8	110.5	-8.4	110.9	-4.1	100.3	-9.1
AC	α_4	114.6	-2.6	109.7	-3.5	90.2	-10.3	87.2	-8.7	80.3	-7.5
AC	β_1	2.5	2.4	1.1	1.2	-3.0	-2.7	0.2	0.1	3.7	1.9
AC	β_2	1.9	1.7	-2.5	-2.1	-1.0	-1.2	-2.5	-1.8	-0.6	1.7
CR	α_1	37.0	-1.7	38.3	1.0	35.4	-3.3	29.4	-5.3	24.1	-10.5
CR	α_2	35.6	-2.5	37.4	0.0	33.4	-2.8	26.5	-7.5	22.0	-11.0
CR	α_3	35.9	-5.6	36.3	-0.2	30.6	-2.9	22.7	-5.6	15.7	-8.3
CR	α_4	37.4	-1.1	37.0	-0.6	30.9	-4.3	22.8	-8.9	18.1	-8.2
CR	β_1	0.3	-1.8	1.6	1.3	-0.9	-2.1	-4.1	-4.3	-4.6	-4.6
CR	β_2	1.5	-0.3	0.4	-0.1	0.7	0.5	-1.0	-0.4	-0.1	-1.0
CM	α_1	19.7	-3.7	22.0	-2.2	20.0	-3.3	18.1	-3.2	15.9	-1.5
CM	α_2	11.3	-3.4	13.9	-1.7	11.6	-3.6	10.1	-2.1	8.8	-0.3
CM	α_3	10.1	-3.2	12.1	-1.8	9.2	-4.1	8.6	-0.9	7.0	-2.9
CM	α_4	14.4	-2.8	15.7	-1.5	11.8	-4.4	11.9	-1.2	9.8	-3.3
CM	β_1	-0.8	-1.4	0.1	-0.4	-2.7	-4.2	0.4	0.1	0.7	-2.2
CM	β_2	0.2	-0.7	0.9	0.8	-2.8	-2.8	1.8	3.5	1.4	1.8

ODS: outcome-dependent sampling; SM: stereotype model; AC: adjacent-category logit model;
CR: continuation-ratio logit model; CM: cumulative logit model.

Table S9: Percent (%) relative error of model-based standard errors and Percent (%) relative error of design-based standard errors of weighted models under ODS from Scenarios H(i), H(ii), H(iii), H(iv), and H(v) with $K = 5$.

		Scenario H(i)		Scenario H(ii)		Scenario H(iii)		Scenario H(iv)		Scenario H(v)	
Model	$\boldsymbol{\theta}$	RE($\hat{\boldsymbol{\theta}}_u$)	RE($\hat{\boldsymbol{\theta}}_w$)	RE($\hat{\boldsymbol{\theta}}_u$)	RE($\hat{\boldsymbol{\theta}}_w$)	RE($\hat{\boldsymbol{\theta}}_u$)	RE($\hat{\boldsymbol{\theta}}_w$)	RE($\hat{\boldsymbol{\theta}}_u$)	RE($\hat{\boldsymbol{\theta}}_w$)	RE($\hat{\boldsymbol{\theta}}_u$)	RE($\hat{\boldsymbol{\theta}}_w$)
SM	α_1	6.6	2.2	9.7	3.1	4.3	-2.0	5.7	-1.0	5.5	-0.7
SM	α_2	16.7	1.1	17.4	0.4	21.4	-0.0	26.4	-1.1	32.1	-6.0
SM	α_3	34.1	0.4	31.6	-0.5	30.2	-2.5	41.1	1.5	42.9	-5.3
SM	α_4	68.1	1.3	64.4	-2.2	61.7	-0.1	68.6	-4.4	67.2	-8.0
SM	β_1	-1.6	2.6	-0.7	2.2	-6.3	-3.8	-1.5	1.1	-4.3	-1.7
SM	$\phi_2\beta_1$	-1.0	2.0	-4.1	-1.6	-5.8	-2.7	0.8	3.7	-5.0	-0.9
SM	$\phi_3\beta_1$	-1.8	1.3	-1.0	1.7	-6.7	-2.8	-0.1	3.5	-2.4	1.8
SM	$\phi_4\beta_1$	-2.6	0.3	-2.9	-0.4	-3.3	-0.1	-1.1	2.6	-2.1	2.0
SM	β_2	0.5	3.7	-3.7	-0.2	-3.6	-0.6	-1.1	2.0	-4.7	-1.7
SM	$\phi_2\beta_2$	-0.8	1.6	-4.8	-1.7	-7.1	-4.0	-0.1	4.5	-5.3	-1.8
SM	$\phi_3\beta_2$	-1.4	0.1	-1.3	1.7	-4.1	-1.4	-1.4	2.8	-6.5	-2.3
SM	$\phi_4\beta_2$	-4.0	-2.2	-4.0	-1.4	-4.8	-1.7	-2.4	2.1	-3.4	0.7
AC	α_1	143.7	-5.4	150.9	-8.6	149.2	-8.0	149.7	-11.0	134.2	-18.0
AC	α_2	133.4	-4.7	145.4	-7.1	147.8	-8.5	150.2	-13.7	135.5	-18.6
AC	α_3	110.9	-4.6	128.4	-5.0	134.7	-8.7	140.8	-10.2	128.8	-19.5
AC	α_4	88.9	-11.5	107.9	-3.4	115.4	-3.9	124.3	-7.0	114.7	-9.9
AC	β_1	-2.9	-2.3	0.2	0.3	-0.4	-0.4	3.4	-0.3	-0.7	-3.6
AC	β_2	-1.9	-0.9	-0.3	0.4	-5.2	-4.0	-1.1	-3.6	-1.2	-2.9
CR	α_1	35.5	0.3	40.0	1.4	40.5	1.6	33.9	-2.7	42.4	-1.0
CR	α_2	33.0	-0.2	38.3	1.5	40.6	1.3	34.5	-1.6	44.6	-0.7
CR	α_3	28.5	-2.2	36.8	0.1	41.5	2.3	35.6	-2.3	47.5	-1.4
CR	α_4	26.3	-5.1	37.1	-0.1	45.3	0.5	40.4	-2.5	54.1	-1.3
CR	β_1	-0.9	-1.9	1.5	0.9	1.7	1.0	-1.8	-0.5	5.7	-1.3
CR	β_2	-2.1	-1.0	1.2	0.8	-2.0	-2.0	-0.7	-1.7	0.7	-0.5
CM	α_1	26.5	2.6	19.4	-4.4	25.2	-0.9	24.2	-0.2	20.2	-1.4
CM	α_2	17.4	3.2	10.6	-4.8	16.3	-0.7	16.0	-0.4	12.9	-0.4
CM	α_3	14.9	3.2	8.2	-4.7	14.6	-0.5	14.9	-0.6	12.6	0.0
CM	α_4	17.6	2.8	11.5	-4.5	18.8	-0.9	19.9	-1.0	18.1	-0.0
CM	β_1	2.7	3.3	-3.0	-3.4	0.3	-1.0	-0.8	-3.0	-2.8	-1.4
CM	β_2	1.9	1.6	0.7	0.5	0.6	1.1	-0.5	2.9	-1.2	-2.4

ODS: outcome-dependent sampling; SM: stereotype model; AC: adjacent-category logit model;
CR: continuation-ratio logit model; CM: cumulative logit model.

Table S10: Percent (%) relative error of model-based standard errors and Percent (%) relative error of design-based standard errors of weighted models under ODS from Scenarios L(i), L(ii), L(iii), L(iv), and L(v) with $K = 3$.

		Scenario L(i)		Scenario L(ii)		Scenario L(iii)		Scenario L(iv)		Scenario L(v)	
Model	$\boldsymbol{\theta}$	RE($\hat{\boldsymbol{\theta}}_u$)	RE($\hat{\boldsymbol{\theta}}_w$)	RE($\hat{\boldsymbol{\theta}}_u$)	RE($\hat{\boldsymbol{\theta}}_w$)	RE($\hat{\boldsymbol{\theta}}_u$)	RE($\hat{\boldsymbol{\theta}}_w$)	RE($\hat{\boldsymbol{\theta}}_u$)	RE($\hat{\boldsymbol{\theta}}_w$)	RE($\hat{\boldsymbol{\theta}}_u$)	RE($\hat{\boldsymbol{\theta}}_w$)
SM	α_1	7.3	-2.4	5.7	-2.3	11.2	3.2	7.6	-0.2	12.3	2.7
SM	α_2	36.8	-2.0	27.6	-2.3	23.8	-2.1	17.0	-2.7	11.0	-3.8
SM	β_1	-1.9	-1.4	-6.1	-4.6	2.3	3.6	-0.5	0.4	3.4	1.9
SM	$\phi_2\beta_1$	-0.7	1.6	-4.8	-2.4	-2.4	-0.3	-2.8	-1.2	-5.0	-4.0
SM	β_2	0.3	0.9	-2.4	-0.4	-3.9	-1.4	-2.9	-0.7	-2.8	-0.7
SM	$\phi_2\beta_2$	0.2	3.1	-1.5	0.7	-2.1	0.9	-4.9	-2.7	-5.4	-2.5
AC	α_1	37.3	-4.0	38.7	-2.4	35.7	-5.2	37.5	-1.9	34.3	-4.9
AC	α_2	28.7	-3.4	32.6	-1.3	27.2	-4.7	29.2	-0.9	22.7	-6.9
AC	β_1	-3.1	-3.6	-0.6	-0.2	-1.9	-2.5	-0.1	0.2	-2.9	-4.5
AC	β_2	-2.2	-0.7	-3.3	-2.9	2.5	3.0	-1.4	-1.2	-1.1	0.1
CR	α_1	20.7	-3.1	15.2	-4.4	14.7	-4.1	12.0	-7.4	14.3	-3.6
CR	α_2	20.5	-1.6	13.5	-4.7	14.5	-3.3	10.8	-7.3	11.4	-4.2
CR	β_1	1.5	-1.6	-3.4	-3.4	-3.0	-2.2	-2.6	-4.0	-2.0	-2.6
CR	β_2	1.2	-0.5	0.5	0.4	-6.0	-5.6	1.9	1.7	0.6	-0.2
CM	α_1	11.1	-3.5	14.3	-1.7	14.1	-1.0	9.3	-4.5	9.2	-0.8
CM	α_2	8.3	-3.7	10.8	-1.2	11.2	-1.1	6.2	-4.8	6.3	-1.1
CM	β_1	-4.4	-4.0	-1.3	-1.1	1.2	1.8	-2.9	-3.3	-0.9	-0.8
CM	β_2	-4.1	-1.9	0.5	1.0	-0.9	-0.3	-3.3	-1.2	-7.7	-5.6

ODS: outcome-dependent sampling; SM: stereotype model; AC: adjacent-category logit model;

CR: continuation-ratio logit model; CM: cumulative logit model.

Table S11: Percent (%) relative error of model-based standard errors and Percent (%) relative error of design-based standard errors of weighted models under ODS from Scenarios M(i), M(ii), M(iii), M(iv), and M(v) with $K = 3$.

		Scenario M(i)		Scenario M(ii)		Scenario M(iii)		Scenario M(iv)		Scenario M(v)	
Model	$\boldsymbol{\theta}$	RE($\hat{\boldsymbol{\theta}}_u$)	RE($\hat{\boldsymbol{\theta}}_w$)	RE($\hat{\boldsymbol{\theta}}_u$)	RE($\hat{\boldsymbol{\theta}}_w$)	RE($\hat{\boldsymbol{\theta}}_u$)	RE($\hat{\boldsymbol{\theta}}_w$)	RE($\hat{\boldsymbol{\theta}}_u$)	RE($\hat{\boldsymbol{\theta}}_w$)	RE($\hat{\boldsymbol{\theta}}_u$)	RE($\hat{\boldsymbol{\theta}}_w$)
SM	α_1	8.3	-0.6	5.6	-1.8	7.5	0.1	8.4	0.3	4.6	-0.8
SM	α_2	30.3	-2.2	30.6	-0.2	31.7	0.9	32.4	0.7	25.9	-1.4
SM	β_1	-0.9	0.5	-5.9	-4.4	-2.6	-0.8	-0.4	0.4	-2.5	-2.0
SM	$\phi_2\beta_1$	-2.0	1.9	-3.0	-0.3	-4.1	-1.8	-1.1	-0.5	-2.6	-1.0
SM	β_2	-2.4	-0.2	-1.7	0.3	-0.2	2.3	-1.2	0.9	-1.7	0.7
SM	$\phi_2\beta_2$	-2.4	1.7	-2.8	-0.1	-2.3	-0.5	-2.0	-0.3	-1.3	0.9
AC	α_1	41.5	-5.6	41.1	-1.3	37.6	-2.5	36.2	-0.6	33.7	-0.1
AC	α_2	32.8	-2.8	33.5	-1.3	29.7	-1.8	25.8	-0.9	21.9	-1.6
AC	β_1	-1.2	-0.9	3.3	3.5	-0.5	-0.1	-1.1	-0.3	0.0	0.1
AC	β_2	5.2	5.4	3.2	3.7	-4.5	-4.0	0.4	1.4	2.6	4.9
CR	α_1	18.4	-0.7	18.2	-2.2	16.2	-3.2	17.7	1.2	13.0	-4.3
CR	α_2	21.4	-1.2	17.8	-2.5	12.6	-4.3	13.0	-0.0	6.0	-6.4
CR	β_1	0.9	0.3	-1.2	-1.6	-3.5	-3.5	-1.5	-0.1	-4.4	-5.0
CR	β_2	-3.8	-3.8	0.8	0.6	1.0	1.4	2.4	2.5	-1.4	-2.3
CM	α_1	14.9	-1.8	19.4	2.6	16.5	0.5	16.9	0.9	7.9	-3.3
CM	α_2	11.9	-1.9	16.4	3.3	12.8	0.4	13.3	0.9	4.5	-2.7
CM	β_1	-1.2	-1.5	3.6	3.8	0.6	-0.6	3.3	2.2	-1.8	-2.0
CM	β_2	-1.1	-1.2	-3.0	-2.7	1.6	1.8	0.2	-0.1	-2.5	-2.8

ODS: outcome-dependent sampling; SM: stereotype model; AC: adjacent-category logit model;

CR: continuation-ratio logit model; CM: cumulative logit model.

Table S12: Percent (%) relative error of model-based standard errors and Percent (%) relative error of design-based standard errors of weighted models under ODS from Scenarios H(i), H(ii), H(iii), H(iv), and H(v) with $K = 3$.

		Scenario H(i)		Scenario H(ii)		Scenario H(iii)		Scenario H(iv)		Scenario H(v)	
Model	$\boldsymbol{\theta}$	RE($\hat{\boldsymbol{\theta}}_u$)	RE($\hat{\boldsymbol{\theta}}_w$)	RE($\hat{\boldsymbol{\theta}}_u$)	RE($\hat{\boldsymbol{\theta}}_w$)	RE($\hat{\boldsymbol{\theta}}_u$)	RE($\hat{\boldsymbol{\theta}}_w$)	RE($\hat{\boldsymbol{\theta}}_u$)	RE($\hat{\boldsymbol{\theta}}_w$)	RE($\hat{\boldsymbol{\theta}}_u$)	RE($\hat{\boldsymbol{\theta}}_w$)
SM	α_1	6.1	-2.4	8.2	0.7	8.6	0.3	7.3	-1.7	11.5	1.5
SM	α_2	24.4	-4.3	30.7	2.1	32.2	-1.1	40.2	-6.1	82.6	-13.1
SM	β_1	-1.7	-0.7	-2.4	-0.7	0.0	0.9	-1.8	-1.2	0.1	1.8
SM	$\phi_2\beta_1$	-0.4	-0.5	-0.2	2.8	-4.5	-1.2	-1.5	2.6	-1.2	1.0
SM	β_2	3.3	2.3	-4.5	-2.3	-3.1	-1.1	-3.6	-1.2	-2.2	-0.8
SM	$\phi_2\beta_2$	2.9	2.9	-3.0	-0.8	-6.0	-3.3	-0.6	3.6	-0.3	3.6
AC	α_1	37.8	-1.3	37.7	-2.9	37.2	-6.0	43.3	-1.8	37.7	-6.8
AC	α_2	29.8	-1.0	29.4	-2.5	29.8	-3.4	37.7	-0.3	34.0	-4.5
AC	β_1	1.8	0.8	-1.1	-0.9	-0.3	-0.2	0.5	0.2	0.7	-0.1
AC	β_2	-5.2	-4.7	-2.0	-1.3	1.1	1.8	-3.2	-2.6	0.2	-0.8
CR	α_1	13.9	-1.8	20.1	0.9	20.5	-0.7	20.1	-1.5	20.0	-0.7
CR	α_2	9.8	-2.6	18.8	0.6	20.9	-1.4	22.4	-1.2	24.2	-1.6
CR	β_1	-4.3	-1.1	-1.5	-1.2	0.1	-0.8	2.7	0.5	4.1	-1.6
CR	β_2	-0.8	0.7	-2.3	-2.3	-0.8	-0.3	-3.4	-4.5	-1.7	-2.0
CM	α_1	8.4	-7.2	14.5	-2.1	12.6	-3.2	13.1	-3.2	9.4	-3.2
CM	α_2	4.4	-6.9	10.4	-2.3	9.7	-3.0	10.7	-3.0	8.5	-2.5
CM	β_1	-3.5	-4.6	-3.2	-3.2	-2.0	-2.0	-2.5	-1.6	-1.9	-0.3
CM	β_2	-1.7	-0.9	-1.8	-1.6	-0.4	0.3	-2.7	-2.5	-3.2	0.0

ODS: outcome-dependent sampling; SM: stereotype model; AC: adjacent-category logit model;
CR: continuation-ratio logit model; CM: cumulative logit model.

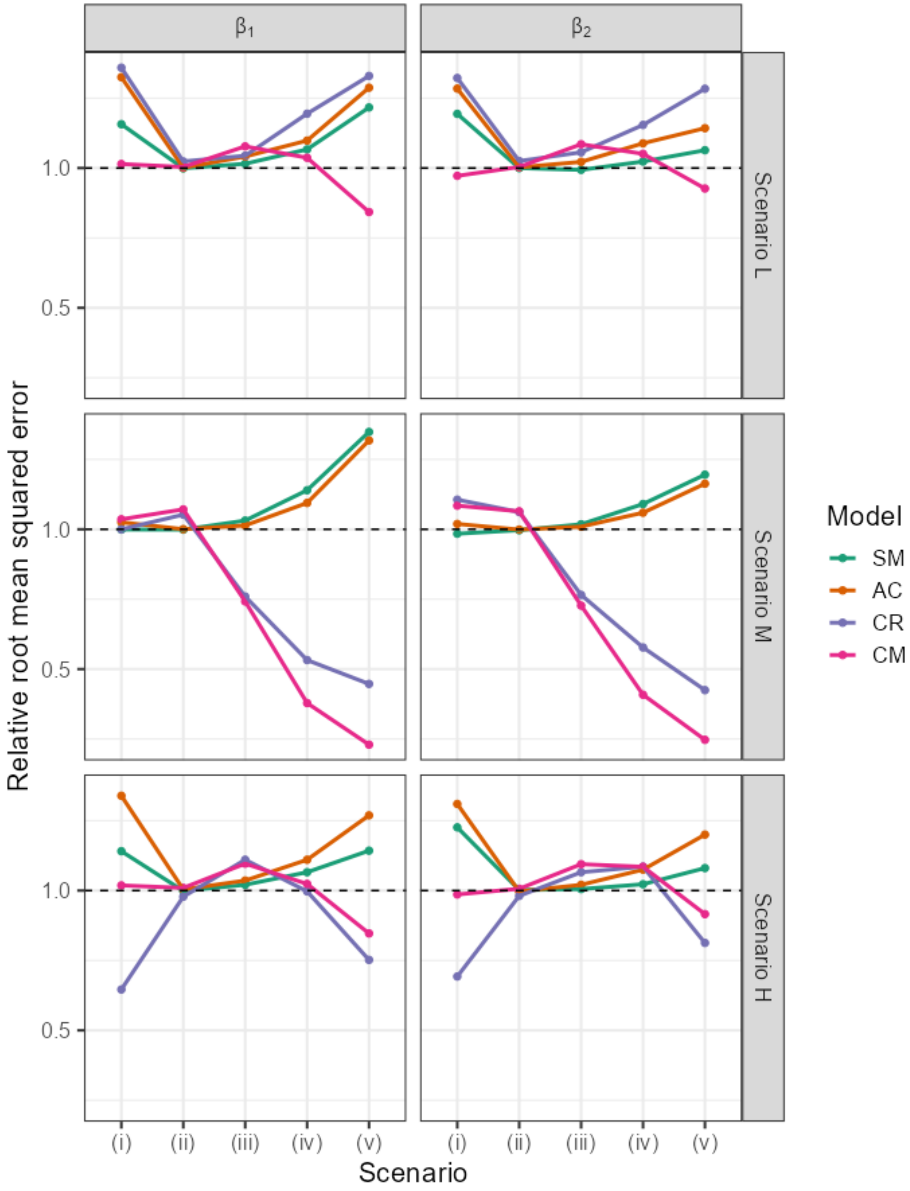

Figure S1: Patterns of relative root mean squared error for β_1 and β_2 from 1,000 simulations comparing unweighted to weighted SM (stereotype model), AC (adjacent-category logit model), CR (continuation-ratio logit model), and CM (cumulative logit model), and across Scenarios L(i-v), M(i-v) with $K = 5$
